# The Regulation of MiTF/TFE Transcription Factors Across Model Organisms: from Brain Physiology to Implication for Neurodegeneration

**DOI:** 10.1007/s12035-022-02895-3

**Published:** 2022-06-04

**Authors:** Francesco Agostini, Rossella Agostinis, Diego L. Medina, Marco Bisaglia, Elisa Greggio, Nicoletta Plotegher

**Affiliations:** 1 Department of Biology, University of Padova, Padua, Italy; 2 Telethon Institute of Genetics and Medicine (TIGEM), Pozzuoli, Naples, Italy; 3 Scuola Superiore Meridionale SSM, Federico II University, Naples, Italy; 4 Department of Medical and Translational, Science, II University, Naples, Federico Italy

**Keywords:** MiTF/TFE, TFEB, Autophagy, Neurodegeneration, Lysosomal storage disorders

## Abstract

The microphthalmia/transcription factor E (MiTF/TFE) transcription factors are responsible for the regulation of various key processes for the maintenance of brain function, including autophagy-lysosomal pathway, lipid catabolism, and mitochondrial homeostasis. Among them, autophagy is one of the most relevant pathways in this frame; it is evolutionary conserved and crucial for cellular homeostasis. The dysregulation of MiTF/TFE proteins was shown to be involved in the development and progression of neurodegenerative diseases. Thus, the characterization of their function is key in the understanding of the etiology of these diseases, with the potential to develop novel therapeutics targeted to MiTF/TFE proteins and to the autophagic process. The fact that these proteins are evolutionary conserved suggests that their function and dysfunction can be investigated in model organisms with a simpler nervous system than the mammalian one. Building not only on studies in mammalian models but also in complementary model organisms, in this review we discuss (1) the mechanistic regulation of MiTF/TFE transcription factors; (2) their roles in different regions of the central nervous system, in different cell types, and their involvement in the development of neurodegenerative diseases, including lysosomal storage disorders; (3) the overlap and the compensation that occur among the different members of the family; (4) the importance of the evolutionary conservation of these protein and the process they regulate, which allows their study in different model organisms; and (5) their possible role as therapeutic targets in neurodegeneration.

## Introduction

The transcription factors of the microphthalmia/transcription factor E (MiTF/TFE) family are crucial for the regulation of different cellular functions [1]. Among the four members of the mammalian MiTF/TFE family, the transcription factor EB (TFEB) is considered the master regulator of autophagy and lysosomal biogenesis because its nuclear translocation, which is controlled by different kinases and phosphatases, triggers the transcription of numerous genes involved in the regulation of this pathway. However, many aspects related not only to TFEB but also to the other MiTF/TFE transcription factors remain to be elucidated. For instance, the different functions of these proteins in different cellular types and tissues, such as the central nervous system (CNS), are still unclear. Indeed, how and to what extent defects in the regulation of the MiTF/TFE transcription factors contribute to the toxic events associated with neurodegenerative disorders are key questions in the understanding of the molecular mechanisms common to these pathologies [2–6].

Multiple other roles have been associated with the members of the MiTF/TFE family, including the regulation of mitophagy [7], lipid catabolism [8, 9], and mitochondrial biogenesis [10]. Although some of their activities may overlap, each homolog of the MiTF/TFE family seems to have a specific pattern of expression and individual functions, which will be addressed in the following sections. Despite the different cellular roles proposed, the most characterized process regulated by MiTF/TFE transcription factors remains to be autophagy.

Autophagy is a crucial process in cellular physiology and is responsible for the degradation of unnecessary and defective cellular components [11]. The autophagic machinery is known to be highly conserved throughout evolution. In fact, the orthologues of many of the genes necessary for this cellular mechanism are ubiquitously expressed in all eukaryotic organisms. Moreover, genetic, morphological, and sequence-based evidence for autophagy confirms the presence of this mechanism in metazoans, in plants, and also in Protista and fungi [12, 13].

**Fig. 1:**
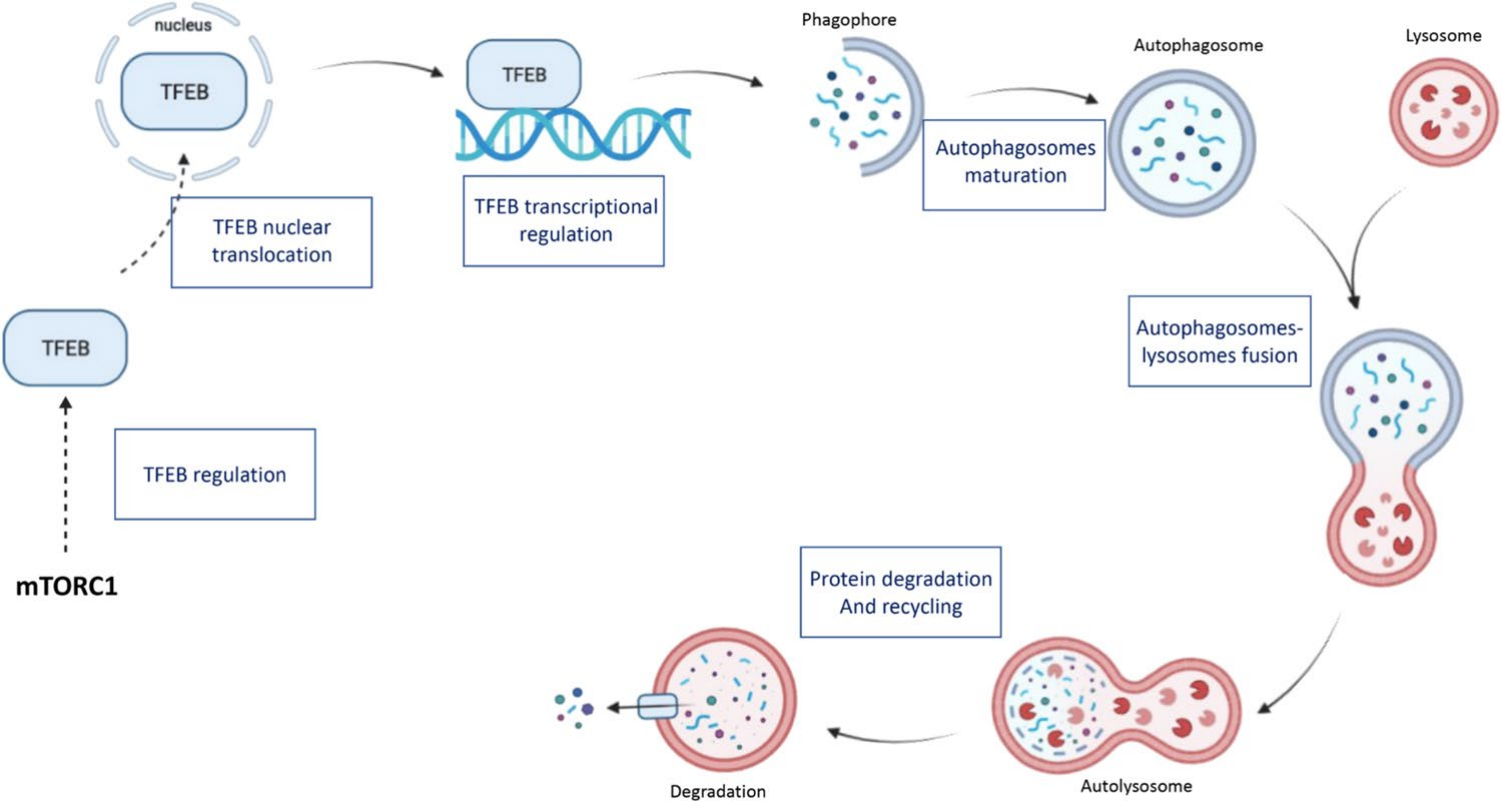
Schematic representation of the different steps of the autophagic process, starting from the most characterized mechanism of TFEB regulation determined by mTORC1 activity, to the degradation of autophagic substrate

Autophagy is a multistep process that determines the engulfment of cytoplasmic components, such as protein aggregates, damaged organelles, and cell debris in double membrane vesicles called autophagosomes (Fig. 1). These vesicles fuse with the lysosomes, membrane bound organelles characterized by an acidic lumen, and lead to the formation of autolysosomes, where the autophagic cargo is degraded [11]. The acidic pH of the lysosomes represents the ideal environment for the hydrolytic enzymes to exert their activity [14]. The products of the autophagic catabolic activity are then recycled back to the cytoplasm to sustain cell homeostasis [11].

Autophagy is ubiquitously performed at the basal level; what differs among cells and tissues is the regulation of the process and the speed of the autophagic flux, which measures the rate of autophagic degradation activity [15].

Autophagic flux is finely regulated by multiple signaling pathways, which are activated by different stimuli, including nutrients, reactive oxygen species and calcium, and by energy imbalance [11, 16]. Autophagy is particularly relevant in the pathogenesis of neurodegenerative diseases, including Alzheimer’s disease (AD), Parkinson’s disease (PD), Huntington’s disease (HD), and in lysosomal storage disorders (LSDs) [17]. All these diseases share a common pathological hallmark, which is the accumulation of aggregated proteins or dysfunctional organelles, such as mitochondria, which are not properly cleared because of defective degradative pathways.

As the MiTF/TFE family is conserved in many different organisms, ranging from mice to fruit flies, and from zebrafishes to worms, their study in these model organisms can provide a better perspective for the interpretation of different findings also in the field of neurodegeneration. The possibility of exploiting different model organisms to characterize the function of the MiTF/TFE family, the processes that they can regulate and their role in neurodegeneration, is also crucial for the identification of new targets for the development of novel therapeutic strategies against neurodegeneration. Finally, the activation of MiTF/TFE transcription factors, particularly of TFEB, can promote the clearance of intracellular waste in both LSDs and more common neurodegenerative diseases [18–20]. This may represent a novel therapeutic strategy to burst lysosomal and autophagic pathways in these disorders by targeting MiTF/TFE proteins.

The role of these transcription factors in autophagy and their link to neurodegeneration have been studied in different models. In this frame, the overexpression of the *Caenorhabditis elegans* orthologue of the MiTF/TFE proteins HLH-30 has been directly associated to autophagic induction and increased lifespan [21]. Coherently, a *Drosophila melanogaster* knockdown model for *Mitf*, the only fly orthologue of these transcription factors, shows autophagic defects and accumulation of autophagic substrate [22]; the activity of Mitf has also been linked to the autophagosomal defects observed in a fly neurodegeneration model of amyotrophic lateral sclerosis (ALS) [23]. Moreover, overexpression of TFEB has been proved to be neuroprotective in a rat model of PD [24]. All these data, and others that will be discussed in detail in the following sections, clearly associate MiTF/TFE transcription factor activity with autophagy and suggest their important function in the field of neurodegeneration. Noteworthy, the knockout of *Tfeb* in mice leads to placental vascularization defects and embryonic death between 9.5 and 10.5 days [25], hampering the possibility to study the effects of *TFEB* knockout on the CNS in a mammalian model.

In this review, we will discuss the function and regulatory mechanisms of the MiTF/TFE family members by comparing their roles in different cell types and tissues, and in different model organisms. This will allow inferring their possible role in different brain cells and regions. We will also describe the role of MiTF/TFE family in neurodegenerative processes and the possibility of targeting these transcription factors to develop novel therapeutics.

By intersecting different aspects of this topic, ranging from function relevant to brain physiology to contribution to neurodegeneration, and by introducing an evolutionary perspective, we envision shedding light on different aspects of this scientific problem. This will also lead to discussing key issues and open questions with the aim of speculating on alternative research lines and on new experimental approaches within this research topic.

## The MiTF/TFE Transcription Factor Family: an Overview

**Fig. 2:**
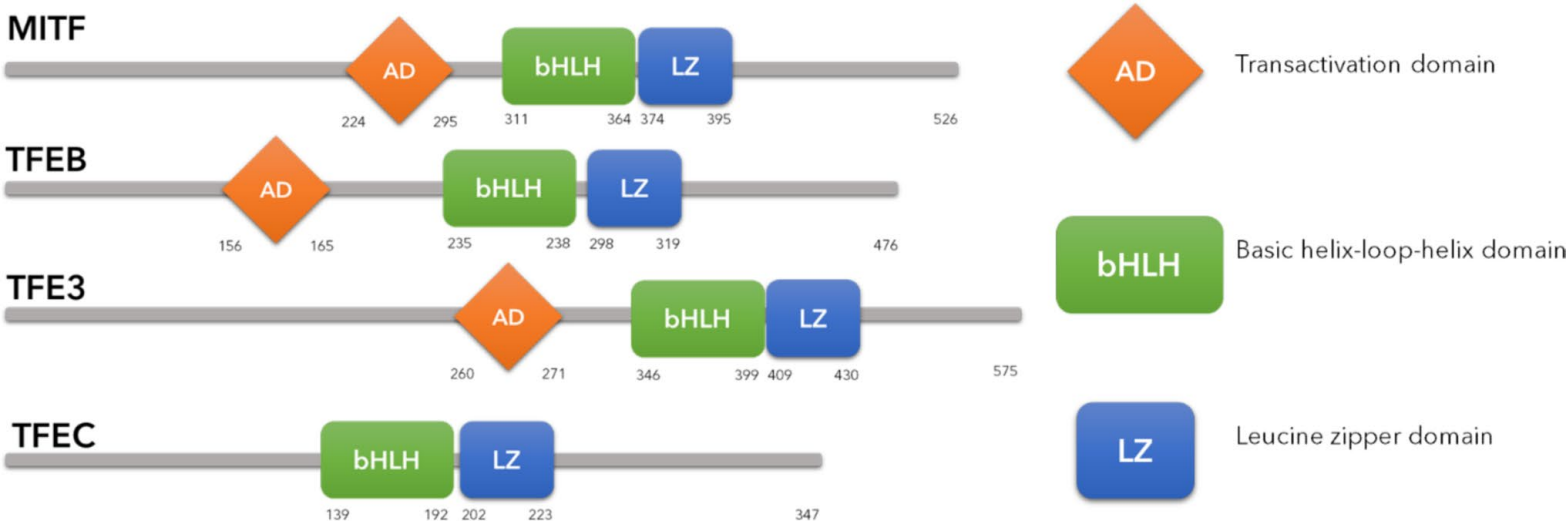
Protein structure of MiT/TFE family members. MiT/TFE family members have high similarities in their sequences: they share basic-helix-loop-helix domain (bHLH) and a leucine zipper (LZ) domain. The activation domain is conserved in TFEB, MITF, and TFE3, but is missing in TFEC

The microphthalmia/transcription factor E (MiTF/TFE) family is constituted in mammals by four members: (i) microphthalmia-associated transcription factor (MITF), (ii) transcription factor EB (TFEB), (iii) transcription factor E3 (TFE3), and (iv) transcription factor EC (TFEC) [4–6]. They share some common structural features: they all contain a basic domain, which is required for DNA binding, and a helix-loop-helix (HLH), and a leucine zipper (LZ) domain, which are critical for dimerization (Fig. 2). TFEB, TFE3, and MITF also contain a conserved transactivation domain, which is crucial for their transcriptional activation whereas TFEC, the most divergent member of the family, lacks this domain and appears to inhibit rather than activate transcription [5, 26, 27] (Fig. 2). The MiTF/TFE transcription factors bind the palindromic CACGTG E-box sequence, which conforms to the CANNTG motif that is recognized by other members of bHLH-zip family transcription factors. Flanking E-box sequences also influence the DNA binding specificity of the HLH/LZ family. The MiTF/TFE transcription factors have been described to prefer the GTCACGTGAC consensus region, named Coordinated Lysosomal Expression and Regulation (CLEAR) motif. Unlike other bHLH-zip transcription factors, the MiTF/TFE family members are also able to bind the asymmetric TCATGTG M-box sequence [28–30]. Sequencing of chromatin immunoprecipitate and mRNA analysis of HeLa cells overexpressing TFEB revealed that, through the binding with the CLEAR motif, TFEB enhances the expression of genes involved in lysosomal biogenesis, in lysosomal membrane formation, in acidification of lysosomes, in lysosomal hydrolases expression, and in the autophagic process [29, 31]. Interestingly, TFEB transcriptional activity is not only limited to the modulation of genes involved in lysosomal homeostasis, but also in the Golgi vesicle transport, protein transport, and mitochondrial homeostasis [31]. These data confirm the close link between TFEB and the other MiTF/TFE proteins and autophagy and underline the crucial role of TFEB in the overall cellular homeostasis.

The MiTF/TFE transcription factors have been shown to form, in vitro, both homodimers and heterodimers with any other family member, and the dimeric form is required for the binding to DNA and the transcriptional activation of target genes. However, they are unable to heterodimerize with other bHLH-zip transcription factors [4, 31]. The X-ray structure of MiTF was obtained using three fragments from the *Mus musculus Mitf* cDNA that were cloned in the pET-M11 vector and expressed in the *Escherichia coli* strain BL21 (DE3) RIL. This structural analysis revealed the presence of a three-residue shift within its ZIP domain, which generates an unusual leucine zipper kink, and is responsible for the specific dimerization of the MiTF/TFE members [31]. Multiple sequence alignment showed that this three-residue shift is conserved among all the MiTF/TFE members, while it is missing in the sequences of the canonical bHLH-zip transcription factors. However, the functional implication of the heterodimer formation has not been fully investigated, except for the MITF-TFE3 heterodimer, which does not appear to be essential for proper functioning, as suggested by MITF and TFE3 redundant roles at least in the development of osteoclasts [32].

## The Regulation of MiTF/TFE Transcription Factors

The regulation of MiTF/TFE transcription factors can occur at different levels. Even though the activity of these proteins is mainly modulated through post-transcriptional modifications and highly depends on their subcellular localization, their regulation can also be performed at the transcriptional level. In this regard, most of the literature available is about TFEB. However, the high degree of homology among the MiTF/TFE members suggests that they may share common regulatory mechanisms. Several transcription factors are known to modulate the expression of *TFEB*: among them, androgen receptors, peroxisome-proliferator activated receptors-α (PPARα) [33], cAMP response element-binding protein (CREB) [34], and Krüppel-like factor 2(KLF2) [35], have been shown to enhance TFEB activity. Moreover, TFEB can also modulate its own regulation through a positive feedback loop [36]. The fact that TFEB is transcriptionally regulated by different transcription factors further suggests the importance of fine-tuning the expression of this protein and underlines that its level and activity are influenced by multiple stimuli. Another level of regulation is represented by alternative splicing. Tissue-specific expression of *MITF*, *TFEB*, and *TFEC* seems to be mediated, at least in part, by alternative transcription start sites, that also allows modulating the activity of the three proteins in different cells. *TFE3* is the only member of the MiTF/TFE family that does not present alternative first exons and is regulated by a single promoter [37].

At the post-transcriptional level, the necessary nuclear shuttling from the steady-state cytosolic localization, that activates TFEB-mediated transcriptional response, correlates with its phosphorylation status and it is positive regulated by de-phosphorylation in key serine residues [2]. The most known member of the family, TFEB, is characterized by several phosphorylation sites within its amino acid sequence. These sites are highly conserved throughout evolution and most of them are found in TFEB orthologues from invertebrates, as *C. elegans*, to humans. Moreover, the other mammalian MiTF/TFE transcription factors present the same phosphorylation sites: the most conserved are the residues crucial for the regulation, such as serine 141, serine 211, and serine 467 [38]. These data suggest that all the MiTF/TFE members may be regulated similarly to TFEB and that these mechanisms of modulation may be relevant for the activity of MiTF/TFE orthologues in different organisms. TFEB is the substrate of different kinase proteins, including extracellular signal-regulated kinase (ERK)2, protein kinase C (PKC)ß, and AKT, also known as protein kinase B (PKB). However, the most important protein involved in the regulation of TFEB is the mechanistic target of rapamycin (mTORC1), which phosphorylates the transcription factor at three serine residues, serine 122, serine 142, and serine 211 [39]. The mTORC1-mediated phosphorylation promotes the interaction between TFEB and 14–3-3 proteins, which sequester TFEB in an inactive state in the cytoplasm. It is important to note that through the binding to Rag (Ras-related GTP-binding) GTPases, both mTORC1 and TFEB are recruited at the lysosomal level, where the interaction occurs [40]. This mechanism is particularly important to respond to environmental cues and occurs in human, mouse, and *Drosophila melanogaster*-derived cells, providing evidence of a mechanism conserved both in mammals and in invertebrates [41, 42]. During normal nutrient conditions, a signaling pathway promoted by the availability of amino acids induces the binding between Rag GTPases and mTORC1 and its consequent activation at the lysosomal surface, where it phosphorylates TFEB [41]. In nutrient deprivation conditions, the calcium-sensitive phosphatase calcineurin dephosphorylates TFEB, thus promoting its nuclear translocation [2]. MITF and TFE3 have also been shown to be similarly regulated by mTORC1, while much less is known about TFEC. Given the high degree of homology with the other members of the family, it is likely that also TFEC is regulated similarly [1].

mTORC1 being the hub of several important cellular processes [43], it is crucial to intensively study its activity and all its regulatory mechanisms to thoroughly understand how TFEB and the other MiTF/TFE proteins are modulated. Nutrient conditions are not the only parameter that affects these mTORC1 and, consequently, MiTF/TFE transcription factor activity. In fact, mTORC1 function may also depend on the activity of AMP-activated protein kinase (AMPK). AMPK is considered the sensor of cellular energetic status and is regulated by different upstream stimuli, such as oscillations in the intracellular calcium levels, alterations in oxygen reactive species, and changes in AMP/ATP ratio. AMPK activation inhibits mTORC1 promoting MiTF/TFE nuclear translocation [44]. Moreover, it has been recently reported that AMPK can directly activate TFEB and TFE3 by phosphorylating a cluster of serine residues in the C-terminus of these proteins [45]. Interestingly, it has been also shown that in mice AMPK activation does not enhance autophagy in neurons, further suggesting that these pathways need to be characterized specifically in different cell types and tissues [46].

These data reinforce the concept of MiTF/TFE transcription factors as crucial players in the maintenance of cellular energy balance and strengthen the hypothesis that their activity is particularly essential in highly energy-demanding tissues, like the brain. Noteworthy, the AMPK phosphorylation sites in TFEB are also present in MITF and TFEC and at least one of these residues is found in the MiTF/TFE orthologues of several organisms, both vertebrates and invertebrates, suggesting that this regulation mechanism may be highly conserved throughout evolution [38]. This data may open the possibility to characterize AMPK-mediated regulation of MiTF/TFE proteins not only in cellular models but also in vivo in different model organisms.

The final regulation mechanism is achieved through the degradation of the proteins. The available data for TFEB show that the transcription factor is degraded through the ubiquitin–proteasome pathway and that the half-life of the protein is about 13.5 h in neuronal-like cells, such as SH-SY5Y [47]. The relevance of the degradation process in the modulation of TFEB activity is confirmed by the fact that proteasome inhibition not only causes the accumulation of the protein, but also promotes its nuclear translocation and, in turn, the increased expression of TFEB target genes [47]. Interestingly, it has been shown that mTORC1 activation enhances the rate of TFEB proteasomal degradation, providing feedback mechanisms through which the mTORC1-mediated phosphorylation of TFEB inhibits its nuclear translocation and promotes also its degradation [48, 49].

## Mammalian MiTF/TFE Transcription Factors

MITF, TFEB, TFE3, and TFEC are the four members of the mammalian MiTF/TFE family. Human and mouse MiTF/TFE proteins share a very high sequence identity (Table 1) and the information currently available on these factors mainly derives from data obtained in mouse models. In the following paragraphs, we will detail the pattern of expression and the main functions of the MiTF/TFE transcription factors. Special care, when possible, will be used in comparing the functions, the localization, and the regulation of the different MiTF/TFE family members.

**Table 1:** Identity and similarity values obtained by PROTEIN BLAST search using the amino acid sequence of human and mouse MiTF/TFE family members

MiTF/TFE transcription factor	Identity (%)	Similarity (%)
MITF	93%	95%
TFEB	93%	95%
TFE3	96%	97%
TFEC	70%	76%

### MITF

In humans, the MITF locus is mapped in the short arm of chromosome 3 and spans 229 kbp, with the promoter region that is highly conserved with mice [26]. *MITF* transcription gives rise to several isoforms that are under the control of alternative promoters: *MITF-A* [50], *MITF-B* [51], *MITF-C* [52], *MITF*-*D* [53], *MITF-E* [54], *MITF-H* [55], *MITF-J* [56], *MITF-Mc* [57], and *MITF-M* [58] (Table 2). These isoforms share the same functional domains (transactivation domain, basic domain, bHLH domain, and LZ domain) but differ in the N-termini, as a result of alternative splicing of exon 1, and display a tissue-specific pattern of expression^.^[37]. MITF is predominantly expressed in melanocytes, osteoclasts, mast cells, macrophages, NK cells, and B cells, and in the heart [59]. Moreover, it is expressed in the CNS. Immunohistochemical analysis of the mouse brain showed that MITF is especially expressed in the olfactory bulb (OB), and in tufted and mitral cells that receive signals from the olfactory neurons and transmit them to the olfactory cortex. MITF protein was not detected in other cell types of the OB, including granule cells or astrocytes [60], in contrast with a previous study based on an RT-PCR analysis showing the expression of *MITF* also in T98G and A-172 human glioblastoma cells [52]. One possible explanation for this discrepancy is that *MITF* expression is much higher in mitral and tufted cells of the OB, allowing the detection of the protein by immunohistochemical analysis only in these cell types [60]. Little is known about the function of MITF in the olfactory bulb. A recent study reported that mitral and tufted neurons from mutant mouse knockout for *Mitf* are characterized by a reduced A-type potassium current (IA) likely because of the decreased expression of the potassium channel subunit KCND3, leading thus to hyperexcitability. Moreover, *Mitf* mutant mice exhibit increased olfactory dishabituation, but reduced the ability to detect the odorant following long-term odor exposure. These findings suggest that MITF plays a key role in olfactory adaptation and intrinsic homeostatic plasticity through the regulation of *Kcnd3* expression. Accordingly, MITF signaling has been demonstrated to upregulate *Kcnd3* expression via an enhancer region located in an intron of *Kcnd3* [6]. It remains to be elucidated the possible importance of MITF in the regulation of KCND3, and therefore of potassium currents in other neuronal types. Worth mentioning is the fact that *Kcnd3* mutations were associated with the neurodegenerative disorder spinocerebellar ataxia type 19 [61, 62], suggesting that MITF impact on *Kcnd3* may also regulate neuronal function in the central and peripheral nervous system.

**Table 2:** Pattern of expression of MiTF/TFE alternative transcripts

Protein name	Protein symbol	Transcripts	Expression	Referee
Microphthalmia-associated transcription factor	MITF	MITF-A	Ubiquitous	[50]
		MITF-B	N/A	
		MITF-C	Different cell types excluding melanocytes	[63]
		MITF-D	Preferentially in RPE cells, macrophages, osteoclasts, and mast cells	[53]
		MITF-E	Mast cells and osteoclasts	[54, 64]
		MITF-H	Ubiquitous	[50]
		MITF-J	Osteoclasts, RPE, and HeLa cells	[56]
		MITF-Mc	Mast cells	[57]
		MITF-M	Melanocytes, melanoma cells, and RPE cells	[65]
Transcription factor EB	TFEB	TFEB-A	Placenta, kidney, lung, and prostate Different tissues	
		TFEB-B	Different tissues excluding liver	
		TFEB-C	N/A	[37]
		TFEB-D TFEB-E TFEB-F TFEB-G	Brain Brain Spleen	
Transcription factor E3	TFE3	None	Ubiquitous with the highest expression levels in placenta, lung, and adrenal gland	[37]
Transcription factor EC	TFEC	TFEC-A	Testis, thymus, trachea, colon, and prostate	
		TFEC-B	Different tissues excluding heart and liver	[37]
		TFEC-C	Kidney and small intestine	

As already reviewed by Haq and Fisher in 2011 [66], MITF is required for many other cellular processes. It mediates the survival of melanoblasts and regulates the expression of genes encoding proteins implicated in the cell cycle in cell invasion by affecting actin cytoskeleton and in DNA damage repair and cell metabolism. In this frame, MITF modulates not only catabolic pathways, like autophagy, but also mitochondrial biogenesis and oxidative phosphorylation [67, 68]. MITF regulation of intracellular metabolism and of actin cytoskeleton is likely crucial not only for neurons, but also for other types of brain cells. In fact, specific types of neurons are known to have increased metabolic demands that make them specifically susceptible to neurodegeneration, suggesting that the MITF role may be particularly relevant in those cases. Similarly, MITF actin cytoskeleton remodeling is critical for neuronal shape and for the regulation of dendritic spine morphology [69]. Defects in neuronal metabolism and in the structure of dendritic spines were associated to different neurodegenerative diseases [70].

Through the binding of the CLEAR motif, MITF is also able to promote the expression of lysosomal and autophagy-related genes. Interestingly, in metastatic melanoma tumors, the lysosomal and autophagy genes under the control of MITF are different compared to the ones regulated by TFEB and TFE3, suggesting a distinct role of MITF [71]. These data support the idea that each member of the MiTF/TFE family may regulate the expression of its target genes in a cell/tissue-specific manner, making it crucial to investigate each of them separately not only in different neuronal types, but also in astrocytes and microglia, when studying their role in neurodegeneration.

In light of the numerous functions attributed to MITF, it is not surprising that mutations in the gene encoding the protein are associated with several pathological conditions. For instance, in the mouse models, MITF mutations induce defects in neural crest-derived melanocyte and retinal pigment epithelium differentiation, osteoclastogenesis, mast cell differentiation, and notch signaling that manifest phenotypically as changes in coat color, small eyes, osteopetrosis, and a reduction in NK cell, B cell, and macrophage numbers [66]. In humans, different heterozygous and homozygous *MITF* mutations are associated with Waardenburg syndrome type 2A and type 4, respectively [72, 73]. Waardenburg syndrome is a neurogenic disorder characterized by the combinations of various degrees of sensorineural deafness and pigmentation abnormalities affecting the skin, hair, and eye [74]. Dominant-negative mutations in MITF are also associated with Tietz syndrome, which is characterized by profound deafness and generalized hypopigmentation [73]. Furthermore, biallelic *MITF* mutant alleles are associated with COMMAD syndrome characterized by coloboma, osteopetrosis, microphthalmia, macrocephaly, albinism, and deafness [75].

### TFEB

Among all members of the MiTF/TFE family, TFEB is the most studied and best characterized since it plays a pivotal role in the regulation of autophagy and lysosomal biogenesis. In contrast with the MITF pattern of expression, TFEB is ubiquitously expressed. Seven alternative 5’ exons of the *TFEB* gene have been identified that originate from seven different transcripts, which encode different TFEB isoforms: *TFEB-A*, *TFEB-B*, *TFEB-C*, *TFEB-D*, *TFEB-E*, *TFEB-F*, and *TFEB-G*. Each transcript displays a different tissue distribution pattern, even though the existence of *TFEB-D* transcript still needs to be confirmed since its expression has not been detected in any of the tissues analyzed by RT-PCR. This could be due to very low expression levels.

TFEB is considered the master regulator of lysosomal biogenesis and autophagy signaling pathways because it induces the transcription of both autophagic/lysosomal-related genes through the binding with the CLEAR motif. This binding determines increased expression levels of the entire network of genes that contain this specific motif (the CLEAR network) [28]. The fact that autophagy is an essential cellular process and that TFEB promotes the expression of the CLEAR network can explain the ubiquitous expression of the protein. Moreover, as autophagic activity can vary between different tissues or cell types, the existence of alternative TFEB transcripts with different expression patterns may account for a very specific regulation of this evolutionarily conserved process. As already mentioned, it is well known that the autophagy-lysosomal pathway (ALP) is key not only for neuronal cells but also for other cell types in the brain, as astrocytes and microglia. In this regard, the contribution of non-neuronal cells to the maintaining of brain homeostasis and their role in neurodegeneration is gaining increasing attention. For example, astrocytes, that are the most abundant glial cells, are involved in the clearance of aggregated proteins and cell debris through the endo-lysosomal pathway. Therefore, the characterization of TFEB activity in non-neuronal brain cells is also very relevant. Increasing the astrocytic or microglial clearance capacity through TFEB upregulation may represent a promising therapeutic strategy for neurodegenerative diseases.

It is already known that TFEB plays an essential role in the tuning of several other basic cellular processes through the regulation of autophagy or lysosomal function in different tissues and cell types. For instance, TFEB-mediated lysosomal biogenesis in differentiated osteoclasts plays a crucial role in bone matrix resorption. Accordingly, mouse osteoclasts lacking TFEB show decreased expression of lysosomal genes, reduced number of lysosomes, and enhanced bone mass [76].

TFEB is also involved in the regulation of lipid metabolism, through a starvation-induced transactivation of *PPARα* and PPAR*α* co-activator 1α (*PGC1α*), which are two key regulators of lipid metabolism during TFEB-mediated starvation. This process has been deeply analyzed in a liver-specific *Tfeb* conditional knockout mouse that displays impaired lipid catabolism and a more severe metabolic imbalance in obese animals. Coherently, TFEB overexpression rescues obesity and associated metabolic syndrome in both diet- and genetically induced obese mice [77].

Through specific gain and loss-of-function approaches in mouse skeletal muscles, the role of TFEB in metabolic adaptation during physical activity has also been emphasized. As a result of exercise, TFEB is dephosphorylated via calcineurin and translocates into the nuclei of myofibers and directly controls glucose homeostasis by regulating the expression of glucose transporters and glycolytic enzymes. Moreover, TFEB modulates the expression of genes implicated in mitochondrial biogenesis, such as mitochondrial transcription factor A (TFAM) and nuclear respiratory factors 1 and 2 (NRF1 and NRF2). It is however unclear whether TFEB activation in response to the exercise depends or not on PGC1α [78, 79].

The overall metabolism of cells seems to be controlled by TFEB function. In neurons, glucose and lipid homeostasis, as well as mitochondrial ATP production, are key in the maintenance of neurons’ capacity to meet the energy demands during neuronal activity. This suggests that if TFEB is defective, also these processes, together with the impairment of lysosomal function, may concur in damaging neuronal function.

The modulation of inflammatory and immune responses is another important function ascribed to TFEB. More specifically, depletion of *Tfeb* in murine macrophages results in a decreased expression and secretion of several pro-inflammatory cytokines, such as tumor necrosis factor (TNF), interleukin-1β (Il-1β) and Il-6, and chemokines including (C–C motif)-ligand 2 (CCL2) and CCL5, after lipopolysaccharides (LPS) treatment [80]. Neuroinflammation is a well-established event that occurs in many neurodegenerative diseases, even if it is still unclear to which extent it contributes to the etiology of the disease and its progression. The findings on TFEB modulation of inflammation would suggest further investigating how TFEB impacts inflammatory mechanisms in brain-resident immune cells.

Overall, TFEB seems to transcriptionally regulate several pathways that are intertwined and that play a role in the maintenance of brain function, making it crucial not only to better understand each of them separately, but also to investigate how they cooperate.

### TFE3

Like TFEB, TFE3 shows a ubiquitous pattern of expression with the highest levels observed in the placenta, lung, and adrenal gland. *TFE3* seems to be regulated by a single promoter since only one transcript for the *TFE3* gene has been identified [58]. Similar to TFEB, TFE3 binds CLEAR elements regulating the expression of genes related to lysosomal biogenesis and autophagy. However, the ability of TFE3 to control the transcription of lysosomal genes is TFEB-independent, suggesting that the relative abundance of TFEB or TFE3 and/or different regulatory mechanisms determine which of them prevails in activating the lysosomal response [81].

Coherently, many functions ascribed to TFE3 overlap with those associated with TFEB. For instance, like TFEB, also TFE3 is involved in the transcriptional regulation of the immune response. Both proteins orchestrate the cellular response to endoplasmic reticulum (ER) stress by upregulating the expression of the activating transcriptional factor 4 (ATF4) or other unfolded protein response (UPR) genes. While under prolonged ER-stress conditions, TFEB and TFE3 activation contributes to cell death by either a direct binding to pro-apoptotic factors promoters, such as C/EBP homologous protein (CHOP) and p53 upregulated modulator of apoptosis (PUMA), or, indirectly, through the regulation of ATF4, which is also involved in the control of CHOP and PUMA expression. These findings suggest that TFEB, TFE3, and ATF4 may play a dual role in cell fate depending on the severity of the stress [82]. Interestingly, TFE3 is also activated in response to Golgi stress and upregulates the transcription of Golgi-related genes [83].

Noteworthy, both ER and Golgi stress or dysfunction are frequently associated with neurodegeneration [84, 85]. Moreover, accumulating evidence links dyshomeostasis of these organelles with alterations in autophagy activity [86, 87], suggesting that it would be interesting to evaluate whether modulating TFE3 activity may affect the onset and progression of neurodegenerative disease, impacting not only ER and Golgi function but also autophagy.

### TFEC

TFEC is the most divergent and least studied member of the MiTF/TFE family. Three alternative 5’ exons of the *TFEC* gene have been identified: TFEC-A, TFEC-B, and TFEC-C, with the latter encoding for a shorter protein lacking exons 2 and 3. Mouse and rat *Tfec* lacks exon 5 that is found only in the human homolog. Human TFEC transcripts have a restricted and distinct pattern of expression [37]. In mice, TFEC expression is restricted to macrophages [88, 89] and mice lacking *Tfec* develop normally. They are viable and fertile, and normally pigmented, have normal eyes and mast cells, and show no osteopetrosis, thus indicating a redundant role of TFEC in myeloid cell development. *Tfec* expression at both the mRNA and protein levels is specifically induced in mouse macrophages by the Th2 cytokine IL-4. In macrophages lacking *TFEC* treated with IL-4, only few genes are affected by TFEC deficiency including G-CSFR (*Csf3r*) gene, which is upregulated to a lesser extent compared to IL-4 treated wild-type macrophages [90]. TFEC remains functionally uncharacterized and no information are available on its role in the nervous systems; thus, further studies to unravel its role in different human tissues/cells are needed.

## TFEB and Autophagy in Neurodegeneration

The demand for basal autophagy differs among cells, and it appears to be particularly crucial in post-mitotic cells, like neurons, whose survival depends upon a strict regulation of cell homeostasis [91, 92]. Moreover, the role of glial cells is also crucial in the removal of extracellular waste and damaged neurons, which makes ALP important also in these cell types in the frame of neurodegenerative processes [93]. This suggests that autophagy and in particular TFEB and the other members of the MiTF/TFE family of transcription factor may be key not only in the development but also in the treatment of neurodegeneration. Further pieces of evidence supporting this idea are discussed in the following sections.

### Autophagy and TFEB Impairment in Age-Related Neurodegenerative Diseases

It is established that autophagy impairments often occur in age-related or inherited neurodegenerative disorders and accumulating evidence suggests a primary involvement of this process in the pathogenesis of many of them, including PD, HD, and AD [94–96]. Several aggregation-prone proteins, such as huntingtin (HTT), α-synuclein (α-syn), amyloid beta (Aβ), and hyperphosphorylated-tau, are eliminated through autophagy [46, 97–99]. Importantly, these proteins can also negatively impact the autophagic pathway, further contributing to their toxicity [100–103].

The link between autophagy and neurodegeneration is further supported by the fact that several genes whose mutations are associated with the familial forms of different neurodegenerative diseases have a role in the autophagic pathway and removal of key autophagy genes in the mouse brain leading to neurodegeneration [104, 105]. Interestingly, the dysregulation of autophagy in neurodegenerative diseases may also present as an increase in autophagy or TFEB activation, as discussed in Section “.TFEB and Autophagy in Neurodegeneration” This suggests that special care must be taken when designing possible therapeutic approaches impacting these mechanisms, in order to preserve autophagy homeostasis, rather than pushing the autophagic machinery without considering possible undesired effects.

Overall, these pieces of evidence suggest that basal clearance of cytosolic waste through autophagy is crucial for preventing the accumulation of cytoplasmic inclusion in neurons, and in astrocytes and microglia, which are involved in the clearance of brain waste via phagocytosis [93]. Therefore, the upregulation of autophagy may have beneficial effects and many research efforts in the field are aimed at finding molecular modulators of this process, possibly in a cell-type specific manner [6, 7].

**Fig. 3:**
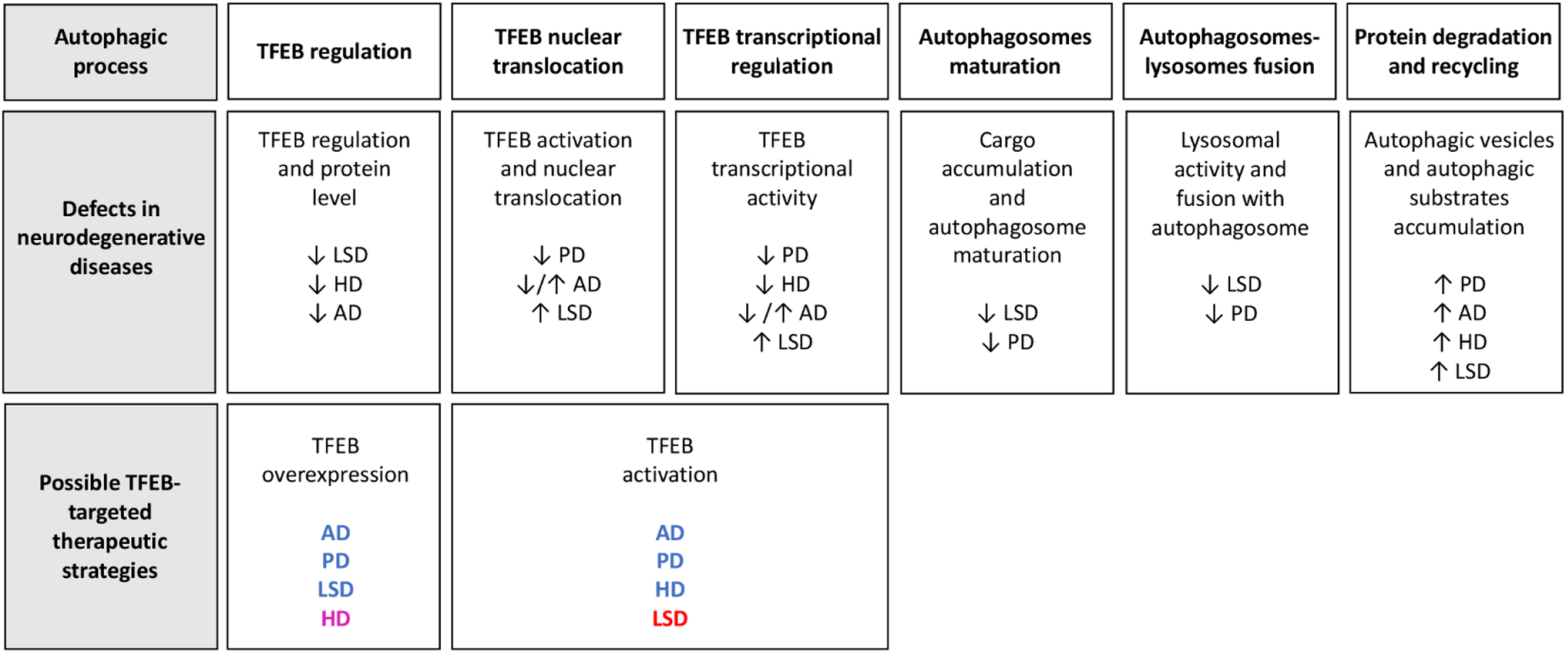
Schematic recapitulation of the defects that characterize different neurodegenerative diseases in every step of the autophagic process (as described in Fig. 1). In the third line of the table are highlighted the possible therapeutic strategies to counteract the progression of these pathologies, like TFEB overexpression and TFEB activation. In blue and red are reported the diseases in which the modulation of TFEB activity has been demonstrated to be beneficial or detrimental, respectively. In magenta the pathology in which the modification of TFEB function may have divergent effects

TFEB, which is by far the most investigated protein in the MiTF/TFE family, shows impaired activity and regulation in many age-related neurodegenerative diseases, further supporting the importance of its role in the maintenance of cellular homeostasis (Fig. 3) [106].

For instance, analysis of postmortem PD midbrains revealed a selective loss of nuclear TFEB. In this frame, TFEB colocalizes with α-syn in Lewy bodies contained in surviving nigral neurons in PD human brains [107]. α-Syn shares structural homology with several regions of 14–3-3 proteins, and 14–3-3 proteins are well-established binding partners of the phosphorylated form of TFEB and prevent its translocation to the nucleus. This homology could lead to the binding between α-syn and TFEB that, consequently, is maintained inactive in the cytoplasm. Indeed, impaired α-syn degradation due to defective autophagy could initiate a vicious cycle leading to non-physiological α-syn-TFEB interaction, which may further amplify ALP dysfunction [107, 108].

The expression of *TFEB* and its target genes was found to be reduced also in the striatum of a mouse model of HD [16]. A reduction of TFEB expression levels and its nuclear localization was also observed in postmortem AD brains as well as in monocytes and lymphocytes from patients with AD [16, 109]. Mouse embryonic fibroblasts from double KO of the AD-associated proteins presenilin 1 and 2 and human AD neurons display higher levels of TFEB phosphorylation compared to the controls, which correlates with TFEB cytoplasmic retention and a decreased CLEAR gene network activity [110].

TFEB nuclear translocation appeared to be negatively affected in a dose-dependent manner in primary microglial cells treated with different concentrations of Aβ [111]. All these data support the presence of an inverse correlation between TFEB nuclear levels and the pathological state, but the picture is more complex and presents also some contrasting results. For example, increased TFEB expression levels were detected in patient-derived fibroblasts carrying the AD-associated presenilin 1 A246E mutation [112]. Another study reported an upregulation of genes within the CLEAR network in brains of presenilin 1, 2 double knockout mice with no changes in TFEB expression level [113]. Analysis of hippocampal CA1 neurons of AD patients microaspirated by laser capture microdissection revealed increased expression levels and nuclear translocation of TFE3, together with an elevated expression of its target genes, while no changes in TFEB levels were detected in the same neurons. Increased TFEB expression levels and nuclear translocation were observed in glia from AD hippocampal tissues, suggesting that TFEB, in this cellular type, may play a crucial role in scavenging aggregated proteins and neuronal debris [114]. Furthermore, these data suggest that TFEB could be upregulated as a compensatory mechanism in certain conditions: if this has a positive effect on the degradation of intracellular waste or contributes to further clump the system is still controversial.

A decline in TFEB levels with age in human immune cells has been recently reported [115], suggesting that it could negatively affect the ALP. This mechanism could contribute to the accumulation of toxic aggregation prone proteins at the early stages of the development of neurodegenerative diseases.

The opposite findings of TFEB being up- or downregulated during neurodegeneration suggest that the extent and length of the neurodegenerative process may impact differently TFEB expression, with an increase in the early phases and a reduction in the late stages of the disease. The same could be hypothesized for TFEB nuclear or cytoplasmic localization. A further degree of complexity is represented by cell type and tissue specificity, which are still understudied, and by the role of the different MiTF/TFE family members in neurodegeneration. The investigation of these aspects in both familial and sporadic forms of these diseases in suitable models may provide interesting clues about the role of these transcription factors in their etiology and further help in the identification of novel therapeutic targets for these disorders.

### Autophagy and TFEB Impairment in Lysosomal Storage Disorders

Another group of diseases in which TFEB and the other transcription factors of the MiTF/TFE family seem to play an important role are lysosomal storage disorders (LSDs). They comprise more than sixty diseases caused by mutations in genes involved in lysosomal function, such as lysosomal hydrolases or lysosomal membrane proteins. About two-thirds of LSDs determine neurological symptoms and are counted among the neurodegenerative disorders [116, 117].

Although these pathologies are characterized by lysosomal defects, in the majority of the cases, they can affect various stages of the autophagic process causing impairments in autophagosome maturation and in autophagosome-lysosome fusion and, eventually, accumulation of undigested material in cells [117]. Because of its role in the regulation of ALP, TFEB has been extensively investigated in the context of LSDs, as a factor that can contribute to the progression of the pathology and as a possible therapeutic target.

As expected, both the activity of TFEB and its nuclear localization together with the autophagic process are affected in several lysosomal disorders (Fig. 3).

Gaucher disease (GD) is the most common LSD and in the most severe cases it determines neurological defects. The pathology is caused by mutation in the *GBA1* gene, which encodes the lysosomal enzyme glucocerebrosidase (GCase), and is characterized by a decrease in the degradation of autophagosome content after its fusion with lysosomes [118]. Lysosomal GCase is responsible for the hydrolysis of the lipid glucosylceramide into glucose and ceramide. When GCase is mutated, accumulation of the substrate and ALP impairment occur in many cell types [118].

Decreased levels of TFEB have been observed in induced pluripotent stem cell (iPSC)-derived neurons from GD patients, probably due to an increased proteasomal degradation of the transcription factor. In the same iPSC-derived model, the instability of TFEB was linked to the hyperactivation of mTORC1 [119]. mTORC1-mediated phosphorylation of TFEB has been shown not only to inhibit its nuclear translocation, but also to promote the targeting of the protein to the proteasomal degradation machinery [48]. Surprisingly, even though mTORC1 activity is increased in GD-derived cells, TFEB was shown to be predominantly localized in the nuclei compared to control cells [118, 119]. These data suggest that another mechanism of TFEB regulation, besides the mTORC1-mediated one, may act in GD cells to stimulate the nuclear translocation and, in turn, the activity of the transcription factor to compensate for the lysosomal defects.

In another study, Sardiello and colleagues investigated the subcellular localization of TFEB in embryonic fibroblast from mouse models of three LSDs: mucopolysaccharidoses types II and III (MPSII, MPSIII) and multiple sulfatase deficiency (MSD). The first two diseases belong to a group of metabolic disorders caused by impairment of lysosomal enzymes required for the degradation of glycosaminoglycans, while the latter is caused by the deficiency in the formylglycine-generating enzyme [120]. As in the case of GD, TFEB was predominantly observed in the nuclei, further supporting the idea that the activation of TFEB is promoted in this type of diseases as a cellular response to enhance lysosomal activity [28].

Despite the nuclear translocation of TFEB observed in several LSDs, this compensatory mechanism does not seem to be enough to counteract the progression of the disease and TFEB activity is not sufficient to properly remove the intracellularly accumulated debris. As observed in GD, it is possible that the stability and accumulation of TFEB are affected by increased proteasomal degradation, resulting in a decreased total amount of the protein. This hypothesis is supported by an experiment performed on myotubes in a mouse model of Pompe disease, another LSD caused by mutation in the *GAA* gene. This disease is characterized by deficiency of the lysosomal enzyme acid α-glucosidase and leads to the accumulation of lysosomal glycogen [121, 122]. While cells treated with the mTOR inhibitor Torin1, which induces a downstream activation of TFEB, failed to rescue the lysosomal phenotype in this model, the overexpression of TFEB in the same model was able to induce cellular clearance, suggesting that in these pathological conditions the amount of endogenous TFEB is not enough to support lysosomal function [121]. Similarly, the overexpression of TFEB in both cellular and mouse models of MSD and MPSIII-A diseases promotes clearance and ameliorates phenotypic hallmarks of these diseases [18].

As previously discussed, also the other members of the MiTF/TFE family are involved in the regulation of the autophagy and lysosomal activity. However, as in the case of the neurodegenerative disorders previously discussed, little is known about the involvement of TFEC and MITF in the onset and progression of LSDs. Recently, it has been demonstrated that the overexpression of TFE3 can induce lysosomal exocytosis and cellular clearance in a model of Pompe disease, suggesting that also this homologue of TFEB can play an important function in the regulation of cell fate in these disorders [123].

### TFEB as a Possible Therapeutic Target in Neurodegeneration

Although the contribution of TFEB to the pathogenesis of neurodegenerative disorders is still under debate, several studies have evaluated the effects induced by the exogenous TFEB overexpression (Fig. 3). In a mouse model of tauopathy, the adenovirus-mediated overexpression of TFEB drastically reduces the levels of the disease marker phospho-Tau 16 weeks post-injection. In this model, TFEB has been shown to participate in the selective elimination of misfolded and hyperphosphorylated tau by promoting the expression of the phosphatase and tensin homolog (PTEN) protein, attenuating neurofibrillary tangles pathology. Moreover, injected mice displayed increased neuronal survival and brain weight, associated with a rescue of behavioral and synaptic deficits [19].

Another study reported decreased levels of tau aggregates in the hippocampus and cortex upon neuron-targeted TFEB overexpression [124], together with attenuated learning and memory skill deficits, in a different mouse model of tauopathy [124].

Extracellular tau is considered to be responsible for the spreading of tau pathology and represents the primary target for tau immunotherapy [125]. Interestingly, TFEB loss of function in PS19 mice, a transgenic mouse line expressing P301S mutant tau, causes a reduction of intestinal fluid tau. The authors proposed a model in which TFEB plays an active role in the secretion of mutant tau via lysosomal exocytosis mediated by TFEB and Transient Receptor Potential Mucolipin 1 (TRPML1) signaling [125]. Accordingly, astrocyte-specific TFEB overexpression in the hippocampus of PS19 mice was able to reduce tau spreading from the ipsilateral to the contralateral hippocampus [126]. A recent study reported that TFEB overexpression in another AD mouse model is responsible for a reduction of the levels of the β-secretase-derived β-amyloid precursor protein fragment C99, which is a precursor of the toxic Aβ peptide. Coherently, the overexpression of TFEB in hippocampal astrocytes contributed to the reduction of Aβ levels in the brain interstitial fluid and of the hippocampal amyloid plaque load [127, 128]. A recent study reported that intracerebral injection of TFEB in the *substantia nigra pars compacta* of a PD rat model overexpressing the human disease-associated A53T α-syn mutant reduced the accumulation and the aggregation of α-syn as well as astrogliosis and prevented the behavioral deficits typical of this PD model [24]. Furthermore, TFEB injection in the striatum of HD^Q175/Q7^ mice reduced the levels of mutant HTT (mHTT) while preserving the levels of wild-type HTT. However, in this model, TFEB overexpression was also accompanied by ER stress and reactive gliosis [129].

In contrast with the later work, another study reported that the co-injection of human TFEB and mHTT in the mouse striatum has no impact on the level of mHTT aggregates even though autophagy appears to be activated. In this case, the accumulation of late autophagic structures seems to impair the global process [130].

Overall, these results seem to indicate that TFEB might represent a promising therapeutic target for the treatment of neurodegenerative disorders. However, it is important to notice that autophagy must be strictly regulated to guarantee the correct homeostasis in each type of cell. Therefore, while a regulated induction of autophagic flux may have positive effects in neurons, the overactivation of this process may be deleterious in other cells. Thus, deciphering the physiological and pathological role of TFEB in the different cell types that constitute the central nervous system will be necessary to develop efficient and safe therapeutic strategies for neurodegenerative disorders.

The possibility of modulating the activity of the MiTF/TFE factors, and in particular of TFEB, has also generated a great interest as a possible therapeutic strategy for LSDs. The role of TFEB as a potential target for the treatment of several LSDs has been largely investigated, and the overexpression of TFEB or its activation through the inhibition of mTORC1 has been reported to be beneficial in the rescue of the lysosome-associated pathological phenotype.

Despite the promising observations in cellular models of LSDs and the first proof of concept in vivo, additional data on the effects of chronic activation of TFEB in animal models is required. Thus, the uncontrolled expression of MiTF/TFE factors is linked to various human rare genetic cancers [131]. In this regard, the most logical approach to promote clearance is the pharmacological activation of TFEB. Using small molecules could allow the modulation of the amplitude and duration of TFEB activity in vivo*.* Also, this approach could be combined to other therapeutic approaches such as enzyme replacement therapy (ERT) or gene therapy.

To evaluate the effect of TFEB modulation in vivo, it would be very useful to take advantage of the different transgenic animal models already used to study LSDs, which are quite reliable compared to the transgenic mouse models for some age-related neurodegenerative diseases. Animal models can provide the opportunity to assess the effects of the constitutive long-term activation of TFEB, for example by evaluating in which way TFEB activation affects the lifespan, the homeostasis, and the function of neurons and of other CNS cell types. They could also help to understand to what extent the modulation of TFEB may be beneficial in the context of LSDs, and how to prevent possible negative effects.

## MiTF/TFE Family Transcription Factors: Putative Therapeutic Targets in Neurodegenerative Diseases and Lysosomal Storage Disorders?

In recent years, several compounds able to modulate TFEB activity have been found to enhance autophagy and lysosomal biogenesis and might have therapeutic potential for the treatment of neurodegenerative diseases and LSD (Table 3). Very recently, a repurposing approach to identify drugs able to ameliorate two subtypes of Batten disease, the most frequent of rare neurodegenerative disorders in children, resulted in the identification of tamoxifen [20]. Tamoxifen ameliorates the phenotype of disease relevant cellular models of CLN3 and CLN7 disease, including neuronal progenitor cells (NPCs) from CLN7 patient-derived induced pluripotent stem cells (iPSC). Also, the treatment with tamoxifen was able to ameliorate the phenotype of a mouse model of CLN7 disease. Interestingly, tamoxifen exerts its action through a mechanism that involves activation of the transcription factor EB (TFEB) [20].

**Table 3:** Modulators of TFEB activity

Name compound	Mechanism of action	Effect	Reference
2-Hydroxypropyl-β-cyclodextrin (2-HPβCD)	Nuclear translocation and consequent activation of TFEB upon treatment	Increased clearance of ceroid lipopigment in late infantile neuronal ceroid lipofuscinosis (LINCL) fibroblasts	[132]
		Increased autophagic clearance of aggregated α-syn in H4 cells stably transfected for the expression of α-syn-EmGFP	[133]
Aspirin (acetylsalicylic acid)	Upregulation of TFEB and increased lysosomal biogenesis via PPARα	Enhanced uptake and degradation of Aβ in primary astrocytes. Reduced intraneuronal Aβ accumulation. Decreased amyloid plaque pathology in 5XFAD mice	[134]
Cerium oxide nanoparticles (nanoceria) coated with N-acetylglucosamine, polyethylene glycol, and polyvinylpyrrolidone	Nuclear translocation and consequent activation of TFEB upon treatment	Promoted clearance of ceroid lipopigment in fibroblasts derived from a patient with late infantile neuronal ceroid lipofuscinosis (LINCL)	[135]
Chlorogenic acid (CGA)	Upregulation of cathepsin D, protein expression induced by the mTOR/TFEB signaling pathway	Promoted lysosomal activity in APP/PS1 mice and Aβ25-35-exposed SHSY5Y cells. Improved spatial memory and attenuated neuron damage in APP/PS1 mice	[136]
Cinnamic acid	Upregulation of TFEB via PPARα	Enhanced lysosomal biogenesis in mouse primary brain cells. Decreased amyloid plaque pathology and improved memory in 5XFAD mice	[137]
Curcumin analog C1	Activation of TFEB and promotion of autophagy and lysosome biogenesis in a mTOR-independent manner	Reduced APP, CTF-β/α, β-amyloid peptides, and Tau aggregates accompanied by improved synaptic and cognitive function in mouse models of beta-amyloidosis, tauopathy, and combined amyloidosis-tauopathy	[138]
		Rescue of cell death in 6-OHDA-induced PD models (SH-SY5Y cells, iPSC-derived DA neurons and mice nigral DA neurons)	[139]
Curcumin derivative (E4)	Activation of TFEB by AKT-MTORC1 inhibition and promotion of autophagy and lysosome biogenesis	Decreased level of overexpressed α–syn in In Neuro2a (N2a) cells transfected with A53T α–syn and reduced cell death in PC12 cells treated with MPP^+^	[140]
Dynasore	Blocking of mTORC1 activity by repressing the lysosomal localization of mTOR, which induces nuclear translocation of TFE3 and TFEB	Enhanced autophagy promotes the clearance of protein aggregates formed by mutant huntingtin	[141]
Fisetin (3,7,3′,4′ -tetrahydroxyflavone)	Activation of TFEB via mTORC1 inhibition	Decreased level of phosphorylated tau in cortical cells or primary neurons	[142]
Flubendazole	Induced TFEB nuclear translocation via mTOR deactivation caused by disruption of dynamic microtubules	Reduction of p-tau in N2a cells	[143]
Genistein (5,7-dihydroxy-3 (4-hydroxyphenyl)-4H-1 benzopyran-4-one)	Impairment of glycosaminoglycans (GAGs) synthesis and enhancement of their degradation. It also alters the expression of genes involved in lysosomal metabolism via TFEB nuclear translocation	Genistein might have beneficial effects for the treatment of lysosomal storage disorders such as mucopolysaccharidoses caused by mutations leading to impaired degradation of GAGs	[144]
GSK3 inhibitor VIII	Activation of TFEB by GSK3 inhibition	Lysosomal clearance of APP in N2a cells stably transfected with the APP-695 Swedish mutation and of its CTF in CHO cells inducibly expressing the APP-CTF	[145]
Gypenoside XVII (GP-17)	TFEB activation by releasing TFEB from TFEB/14–3-3 complexes	Elimination of AβPP, Aβ40, and Aβ42 in APP695swe cells. Formation of Aβ plaques in the hippocampus and cortex of APP/PS1 mice is prevented and spatial learning and memory are restored	[146]
Ibudilast	Enhanced TFEB nuclear translocation by inhibiting mTORC1 activity	Increased clearance of disease-linked TAR DNA binding protein (TDP-43) and superoxide dismutase 1 (SOD1) protein aggregates in cells transfected with corresponding mutated forms. Protective effect of TDP-43-induced cytotoxicity in motor neuron-like NSC-34 cells	[147]
Ouabain	Activation of TFEB via inhibition of the mTOR pathway	Reduced accumulation of p-tau in GFP-TauP301S-overexpressing SH-SY5Y, in primary cortical neurons, in a *Drosophila melanogaster* tau model and in TauP301L mice. Ameliorated memory defects in in TauP301L mice	[148]
Pseudoginsenoside-F11 (PF11)	Induced TFEB nuclear translocation by suppressing mTORC1 activity	Increased degradation of oligomeric Aβ in cultured microglia	[149]
Threalose	Inhibition of Akt which in turn activates TFEB independently of mTORC1	Enhanced clearance of lipopigments and reduced neuropathology in a mouse model of Batten disease. Promoted cellular clearance in fibroblast derived from patient with Batten disease	[150]
	Rapid enlargement and transient permeabilization of lysosomes leading to calcineurin activation and subsequent TFEB dephosphorylation and nuclear translocation	Promoted clearance in mouse motoneuron-like hybrid cell line (NSC34) of polyQ-containing androgen receptor, TDP-43, and SOD1 mutated forms	[151]

The idea of enhancing autophagy to counteract neurodegeneration has been explored by many [152] and in most cases the preferred molecular target for the proposed therapeutic strategies is mTOR, particularly using specific mTOR inhibitors. Despite their efficacy in certain models, they have also proved to have limited capacity of impacting neurodegenerative diseases in certain clinical trials. We suggest that this may be associated to the fact that mTOR regulates not only TFEB and its downstream pathways but also many other targets, and this could be an issue when proposing a therapeutic approach for a chronic progressive disease. Nevertheless, TFEB is a non-canonical substrate of mTOR and can be activated by inhibitors that impact Rags pathways but not on canonical mTOR substrate, such as the ribosomal protein S6 kinase (S6K). For example, in a recently published paper, the mTOR inhibitor fluoxetine was identified as a possible corrector of neurodegeneration in MPS-IIIA via TFEB activation in a Rag-dependent manner [153]. This suggests that careful evaluation of mTOR inhibitors should be performed before moving them towards tests in pre-clinical models for neurodegenerative diseases or to clinical trials. Or, even better, specific therapeutic strategies targeting TFEB should be identified and tested.

Another interesting aspect to evaluate is the fact that research is mainly focused on TFEB among all MiTF/TFE family members, but even though MiTF/TFE transcription factors display some functional overlap, it remains to be established to which extent they have common functions, whether they are complementary or differ, and which factors orchestrate their interplay. Therefore, despite the promising perspective to fight neurodegenerative diseases and LSDs by enhancing autophagy/lysosomal biogenesis via TFEB modulation, a better understanding of the factors that regulate TFEB activity as well as the interplay between TFEB and the MiTF/TFE transcription factors is strongly required. This would ensure a safe development of targeted therapies for the treatment of these diseases. In fact, enhancing autophagy and lysosomal activity may be beneficial for the treatment of neurodegenerative diseases and LSDs but could have adverse effects. For example, it is quite well established that altered regulation of MiTF/TFE proteins can be linked to cancer development. *MITF* gene amplification was found in in 20% of melanomas. Translocations and rearrangements of *TFE3* and *TFEB* are associated with a rare subtype of kidney cancer termed translocation-renal cell carcinoma (tRCC) and alveolar soft part sarcomas (ASPS), a rare lung cancer variant [154]. A recent study reports that TFEC is expressed at higher levels in ovarian cancer tissues, compared to normal tissues, and correlates with malignant progression and poor survival for ovarian cancer patients [155]. Moreover, increased TFEB expression is found in glioblastoma patients and contributes to the glioblastoma resistance to chemotherapy. In fact, drug-mediated inhibition of TFEB expression and oligomerization can enhance glioblastoma cell sensitivity to conventional chemotherapeutic agents [156]. All these observations deserve attention, and therefore a comprehensive characterization of the potential deleterious effects of uncontrolled expression of MiTF/TFE3 factors in vivo. We must consider that alterations to the regulation of MiTF/TFE transcription factors are accompanied by the hyperactivation of other key pathways involved in tumorigenesis and cell proliferation [131, 157]. Therefore, we can expect that controlled pharmacological activation of MiTF/TFE proteins will recapitulate the pathological features of MiTF/TFE-driven cancer.

Overall, given that (1) autophagy and TFEB translocation may already by overactivated in certain neurodegenerative diseases or LSDs to compensate for the defective mechanisms already in place and to remove undigested cellular waste, (2) uncontrolled boosting of autophagy may impact the overall cellular homeostasis, and (3) overactivation of TFEB may trigger downstream pathways other than ALP, the final goal of novel therapeutic strategies would be to restore the homeostatic regulation of these processes and the homeostasis of the MiTF/TFE transcription factors, rather than promoting their uncontrolled activation.

## What Can We Learn from the Study of MiTF/TFE Family in Non-mammalian Organisms?

**Fig. 4:**
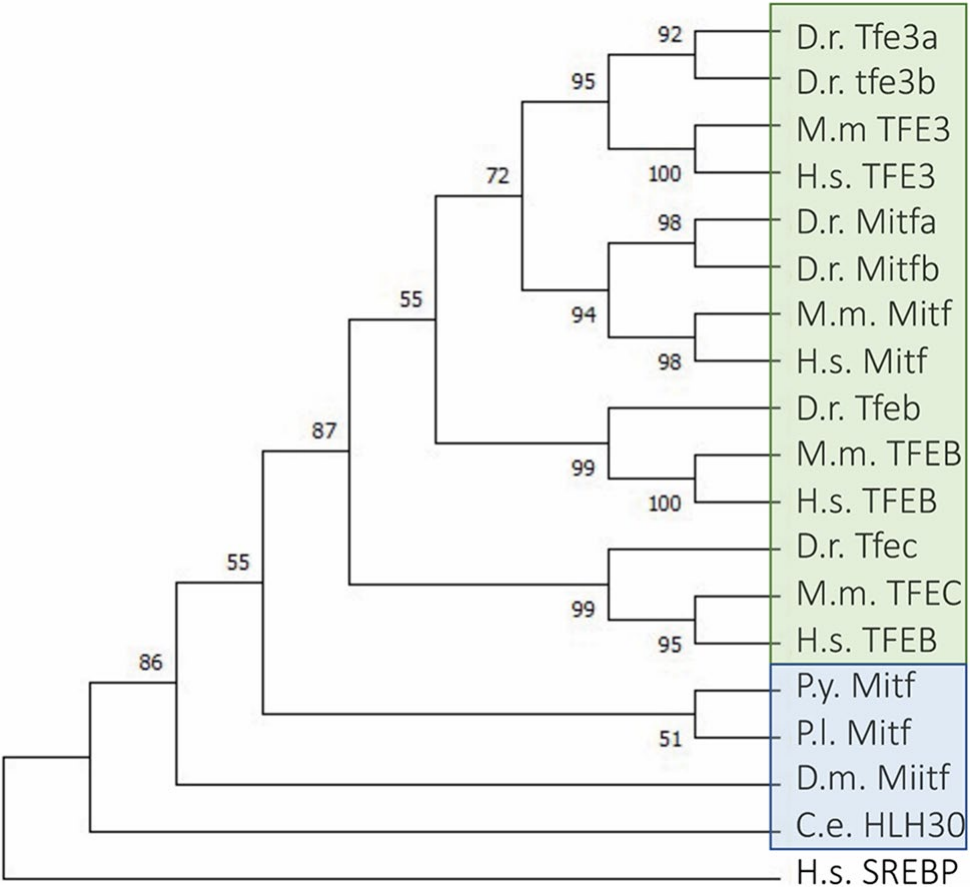
Phylogenetic tree of the MiTF/TFE family of transcription factors. At every node, the bootstrap values are shown. In green and in blue are highlighted vertebrate and invertebrate organisms respectively. *Homo sapiens* Sterol Regulatory Binding Protein (SREBP) has been used as an outgroup protein to root the tree. H.s, *Homo sapiens*; M.m, *Mus musculus*; D.r, *Danio rerio*; P.y, *Patinopecten yessoens*; P.l, *Paracentrotus lividus*; D.m, *Drosophila melanogaster*; C.e, *Caenorhabditis elegans*

**Fig. 5:**
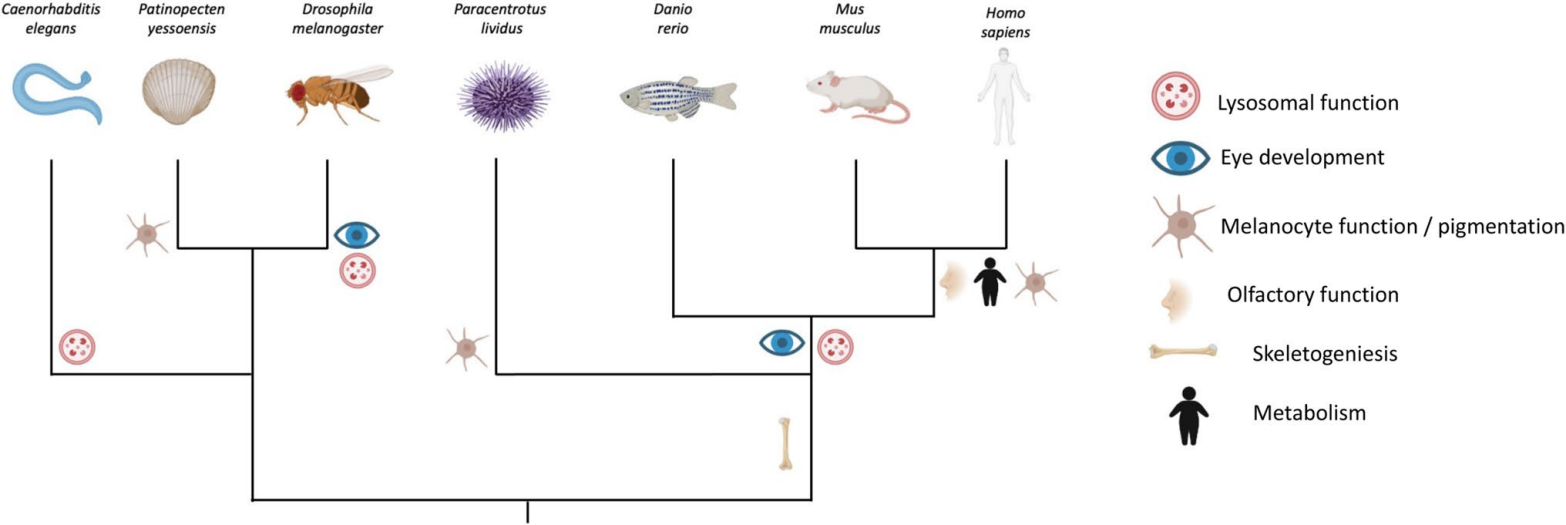
The most important functions ascribed to the MITF/TFE transcription factors are reported in the phylogenetic tree of the species. The position of a specific function in the tree shows when it appeared during evolution. Autophagy and lysosomal activities are the most conserved functions: sea urchins and molluscs are the only organisms in the tree in which this function has not been reported; however, it is highly probable that this function is common to all the organisms and is present in the progenitor of all these animals. Mitf/TFE transcription factors have been associated also to pigmentation and eye development in different organisms. Skeletogenesis seems to be a common function to all deuterostomes. Some peculiar functions, such as the role in the olfaction and the control of metabolism, have been described only in mammals so far

Non-mammalian model organisms can have a role in the implementation of our current knowledge on MiTF/TFE transcription factors. All MiTF/TFE family members are conserved in vertebrates, while invertebrates have only a single MiTF orthologue (Fig. 4). Considering the high degree of conservation of the entire autophagic machinery, also organisms that are phylogenetically distant from humans can be exploited for the in vivo characterization of these proteins and these pathways (Fig. 5). These animal models can give information on unclear or uncharacterized aspects that would otherwise be difficult to study in humans or mice. Every animal model is characterized by specific features that provide unique advantageous tools to understand in detail the activity and the complex mechanisms of regulation of MiTF/TFE transcription factors. For example, the transparent body of worms and zebrafish larvae allows exploiting these organisms for the in vivo visualization of these proteins to study their intracellular localization and movements across the cell compartments. The complex behavioral features of fruit flies may be of great interest for the evaluation of phenotypes associated to the modulation of MiTF/TFE transcription factors activity to study short/long-term collateral effects (if any) and to perform rescue experiments. In addition, exploiting invertebrate models may allow the analysis of a high number of individuals that could be highly valuable for the screening of drugs or compounds that modulate MiTF/TFE protein activity. These simpler organisms are usually easier to be genetically manipulated; they allow the possibility to generate animals carrying specific mutations or useful constructs for imaging that help the understanding of the role of these transcription factors. These are only few of the reasons why the use of non-mammal animal models in the research may be worthwhile. The choice for the proper organism should be based on the questions the researchers want to assess. Moreover, the exploitation of different models to answer the same biological question may be crucial to get more informative results, and to increase the soundness of the data and the value of the research.

The following paragraphs will describe the current knowledge about MiTF/TFE transcription factor in model organisms other than rodents, by focusing, when possible, on phylogenetic and mechanistic aspects.

### Caenorhabditis elegans

The nematode *Caenorhabditis elegans* is one of the most used model organisms in biology [158]. Several features of *C. elegans* make this animal an advantageous model for biology research. First, it is relatively easy to produce transgenic worm lines that overexpress or lack a gene of interest; it is possible to limit the expression of genes to specific cell types and to study protein activity by tagging them with fluorescent probes. Several studies characterized TFEB activity in worm lines lacking the *C. elegans* TFEB orthologue gene or studied its subcellular localization by expressing the protein tagged with a GFP [159–161]. It is also possible to assess autophagic activity in worms through the expression of specific construct for the analysis of autophagic flux [162]. Second, the small size of this organism and the possibility of analyzing many individuals allow performing large-scale screens. While its nervous system is relatively simple compared to that of other model organisms, several cellular and molecular pathways are well conserved. Moreover, about 70% of genes linked to human diseases have orthologues in worm genome. For these reasons, this animal is considered a good model for the neurodegeneration research [163].

A functional orthologue gene of human *TFEB* has been found in *C. elegans*, called *hlh-30*, which shares high homology to the human protein in both the DNA-binding and activation domain. The protein HLH-30 is the only member of the MiTF/TFE family, which is present in *C. elegans* and, like the human orthologue, it modulates autophagy and lysosomal function [164].

Interestingly, also the regulation of HLH-30 protein seems to be conserved, with the worm transcription factor that is modulated via post-transcriptional modifications, in a similar manner to its mammalian orthologues. In fact, the silencing of mTOR has been demonstrated to enhance the HLH-30 nuclear localization and to increase the expression of several genes that are the Nematoda orthologues of the human TFEB targets, including autophagy-related genes [159].

Moreover, the *C. elegans* HLH-30 downstream genes are characterized by the presence in their promoter region of an E-box sequence that overlaps with that of the CLEAR motif, further confirming the conservation between the HLH-30 and its mammalian orthologues. This region in *C. elegans* is crucial for the specificity of HLH-30 binding to DNA [159, 164].

### Drosophila melanogaster

*Drosophila melanogaster*, commonly known as fruit fly, is one of the most used model organism in biology and in biomedical research [165]. In particular, it is a powerful organism to model the physiology and pathology of the brain, mainly because it can be genetically manipulated and because many genes and molecular pathways are conserved between humans and flies [166]. Synapse formation, membrane trafficking, and neuronal communication are a few examples of processes that are similar in flies and in more complex organisms [167]. In addition, *D. melanogaster* brain is developed enough to promote elaborated behavioral features, but it is still small enough and relatively simple, thus allowing the detailed analysis of its structure and functions.

The genome of *Drosophila melanogaster* contains a singl*e Mitf*, which shares several features and functions with its mammalian orthologues, providing evidence of high conservation of this gene throughout the evolution. Interestingly, *Mitf* is not phylogenetically closer to the mammalian *MITF* than to *TFEB*, suggesting that the ancestral gene underwent multiple duplication events after the separation of the invertebrate to the vertebrate lineages [22]. The presence of only one gene in flies and the high degree of conservation with the four mammalian MiTF/TFE family members make the fruit fly an advantageous organism to study its physiological role in a simplified model.

Like the mammalian transcription factors of the MiTF/TFE family, *Drosophila melanogaster* Mitf can regulate gene expression through the binding to the DNA. The conservation of the DNA binding region in the protein suggests that the fly transcription factor recognizes the same target domain of its mammalian orthologues. This DNA region is represented by the already described CLEAR motif, which is crucial for the binding specificity to the DNA. The conservation of the CLEAR motif in *Drosophila* has been confirmed in the promoter region of genes whose expression resulted significantly upregulated by overexpression of Mitf protein [22, 23, 168].

In fruit flies, Mitf has been shown to play a role in the transcriptional regulation of lysosomal biogenesis, in autophagy, and in the catabolism of lipids, further supporting a functional similarity with its mammalian orthologues TFEB and TFE3 [22]. Moreover, a reduction in Mitf activity leads to an impairment of autophagic flux with accumulation of autophagy substrates, such as polyubiquitinated proteins and dysfunctional mitochondria. Mitf determines the cellular response to starvation, which is well known to activate autophagy. Upon starvation, Mitf upregulates lysosomal biogenesis and several autophagy-related genes, especially those involved in the formation and maturation of autophagosomes [22, 168]. In this frame, mTOR has been shown to negatively modulate Mitf activity, and also in flies the treatment with Torin1, a specific inhibitor of mTOR, is able to induce the activation and the nuclear translocation of Mitf [22]. Interestingly, the similarity goes further at the structural level. It is well established that Ser142 and Ser211 are the residues of human TFEB that are phosphorylated by mTORC1, inhibiting TFEB activity. Corresponding serine residues, Ser240 and Ser346, respectively, are also present in the fly orthologue.

Besides its role in autophagy, *D. melanogaster* Mitf is involved in eye development, a function that is performed by MITF in mammals. More specifically, Mitf is expressed in the eye-antennal imaginal disc during the second and third larval stages of the fruit fly and the expression of a dominant negative *Mitf* mutant impairs the correct development of the eye [168].

Overall, the data concerning *D. melanogaster* Mitf suggest that the protein function is highly conserved and that the distinct roles described for the mammalian proteins might coexist in the unique fly orthologue. This makes *D. melanogaster* a perfect asset to test drugs able to modulate Mitf activity in a cost-effective but valuable model organism, and before moving towards more complex organisms.

The presence of a unique *Mitf* orthologue both in *C. elegans* and *D. melanogaster* points at them as good organisms to study this protein in a simple model in which the presence of only one protein of the MiTF/TFE family removes the potential uncertainty derived from the redundancy. On the other side, this hinders the possibility of studying the interplay among the different MiTF/TFE family members, which may be crucial for the understanding of the molecular mechanisms that regulate the downstream pathways of these transcription factors. Under these circumstances, it may be worthwhile to exploit more complex model organisms.

### Danio rerio

Among the vertebrates, *Danio rerio*, commonly known as zebrafish, is one of the most studied model organisms [169]. As a vertebrate, it is evolutionary closer to humans than invertebrate models and present several advantages over other vertebrate organisms, such as the high rate of fecundity, the external fertilization, and the fact that in the first developmental stages the organism is transparent and allows to visualize internal structures and tissues by in vivo imaging [169]. Furthermore, in the neurodegenerative field, zebrafish is largely used because its brain organization shows high similarities with human brain, with specific brain regions of zebrafish that are highly conserved and can be related to mammal brain [169]. Genomic analyses have demonstrated that zebrafish and other teleost species underwent an event of gene duplication that may have occurred at least 100 million years ago and determined the presence of approximately 20–30% of an extra complement of genes in their genome. This is probably the reason for the presence in zebrafish of six genes belonging to the MiTF/TFE family. Besides *tfeb* and *tfec*, it also presents two orthologues of the mammalian *MITF* (*Mitfa* and *Mitfb*) and two orthologues of the mammalian *TFE3* (*Tfe3a* and *Tfe3b*). Since often-duplicated genes are characterized by tissue-specific expression or by the activation in precise developmental stages, the research may take advantage of the duplication of the genome to study in detail the role of the protein of interest in different cell types or at different times. This feature seems to apply to the proteins Mitfa and Mitfb that share high homology in their sequence, with the differences mostly located in their amino and carboxy termini. Interestingly, Mitfa seems to correspond to the mammal melanocytic “M” isoform, whereas Mitfb shares the highest homology with the mammalian “A” isoform. Moreover, the zebrafish *Mitf* genes have restricted expression profiles that approximate the localized expression of their mammalian orthologue [170].

Regarding the role of tfeb, the major functions of this transcription factor are conserved. In zebrafish, as in mammals, it controls the network of genes involved in lysosomal biogenesis and autophagy. Moreover, similarly to its mammalian orthologue, tfeb activation is regulated by mTORC1 and Rag-GTPases. Like in mouse, tfeb can repress in zebrafish the process of myelination during the development of the CNS. More specifically, tfeb has been shown to upregulate several of its target genes in the oligodentrocytes, leading the authors to speculate that the activity of tfeb may impair the trafficking of endo-lysosomal organelles to the membrane and the synthesis of lipids, two crucial processes for the membranous myelin sheath. As the activity of tfeb might disrupt the process of myelination, it appears to be specifically repressed by mTORC1 and other inhibitory kinases during myelination [171]. This function of tfeb, although poorly investigated, may be crucial for neuronal physiology and could be very relevant in the context of neurodegeneration. Thus, it would be very worthwhile to investigate whether this activity of tfeb is conserved in other models and in mammal and understand how it impacts brain homeostasis.

In comparison to *Mitf* and *Tfeb*, which are the most studied and characterized genes among the members of the MiTF/TFE family in zebrafish, much less is known about the two *tfe3* genes. *Tfe3a* has been described to encode a protein of 539 amino acids that shares about 50% of homology with the human *TFE3*. As observed for the other members of the family, the most conserved region of *Tfe3a* is the helix-loop-helix/leucine zipper region. Like the human orthologue that has been shown to regulate immunoglobulin expression, *tfe3a* in zebrafish is present in the ventral mesoderm, which gives origin to blood cells, suggesting a possible functional conservation of this gene between different organisms [170].

*Tfe3* genes seem to be co-expressed with *mitf* genes in several tissues. In fibroblast cell cultures, the two genes share comparable activities, suggesting a possible redundant role. Nevertheless, in mitf knockout zebrafish models, Tfe3 has been demonstrated to support very inefficiently the role of Mitf [170], indicating that, in vivo, the different members of the MiTF/TFE family exert different roles being only partially redundant at the functional level [170].

In zebrafish, *Tfec* is the less characterized member of the Mitf family. The protein has been proposed to be a key regulator of zebrafish embryonic hematopoiesis, the process responsible for the formation of all types of blood cells from hematopoietic stem cells (HSCs) [172]. How and if this has a role in the nervous system, at least in this model, remain to be elucidated.

### Other Organisms

Beside the most common and studied animal models, orthologues of the MiTF/TFE family of transcription factors have been found in other organisms. Even though these organisms are considered irrelevant in biomedical research, they may inspire the study of alternative pathways or regulatory mechanisms in more conventional models.

Among the organisms in which an orthologue of Mitf has been described, *Paracentrotus lividus* is a sea urchin that has an important phylogenetic position because it belongs to the phylum of Deuterostome, like vertebrates. Given the conservation of many important molecular pathways, the study of Mitf in *Paracentrotus lividus* may provide interesting information about the role and the signalling pathways of this transcription factor.

Pl-Mitf protein is characterized by all the functional domains of the MiTF/TFE protein family, including the DNA binding domain and the bHLHzip domain. However, in this domain, only four out of five canonical leucine residues are observed and two of them are conservative substitutions. This imperfect leucine zipper has also been found in *D. melanogaster*. Among the phosphorylation sites of Pl-Mitf, some of them are conserved, further providing evidence of a possible common pattern of regulation of this protein. Mitf has been found in the pigment cells of the sea urchin, coherently to the role of the mammalian orthologue in melanocyte. Moreover, while MITF in mammals is an important transcription factor in osteoclasts, Pl-Mitf is expressed in the presumptive mesenchymal cells (PMC) that are progenitor cells of the sea urchin larval skeleton. Some studies have highlighted similar features between PMC and osteoclasts: both cell types are involved in the skeleton development, have migratory capability, and can form multinucleated syncytia. These data may suggest an unknown role of Pl-Mitf in the skeletogenesis of sea urchin [173].

Given the well-established role of MITF in the pigment cells, another group of organisms may be interesting to study the function of this gene. In fact, molluscs are characterized by a vast pattern of colors, mainly in shells. The yesso scallop, *Patinopecten yessoensis*, is a large group of molluscs that live in the bottom of the northwestern Pacific Ocean. The genome and the transcriptome of Yesso scallop have been widely studied and a unique orthologue of *MITF* (*Py-Mitf*) has been found in this organism. It shares less homology with vertebrate organisms, highlighting the fact that the evolution of this gene is consistent with the species taxonomy. This gene is formed by eight exons, in contrast with the mammalian MITF that is organized into nine exons. As observed in other organisms, the most conserved region of the *Py-Mitf* gene is the bHLH-LZ motif [174]. Shell color is determined in these organisms by the presence of biological pigments, like melanin, carotenoids, and tetrapyrroles, but the mechanisms that underline these features are poorly understood. Melanin biosynthesis is initiated with tyrosine oxidation and tyrosine in mammals is known to be positively regulated by MITF, which has been reported to be a master regulator of melanogenesis. Interestingly, in yesso scallop, the expression of *Py-Mitf* has been shown to correlate with the shell color, further confirming the high degree of conservation of these genes among different organisms. Moreover, the higher level of Py-Mitf mRNA was detected in the mantel, the organ involved in shell color formation. Notably, even though the two valves of the same organism usually are characterized by different colors, no difference in the level of Py-Mitf expression was detected between the right and the left mantels. This result may indicate that Py-Mitf is strictly regulated, and a different regulation process may modulate the shell color in the same animal. Reports about Mitf involvement in autophagic regulation in these organisms are lacking; however, given the high conservation of autophagy throughout evolution and the similarities between mammalian MITF and the invertebrate orthologue, it is highly probable that this transcription factor may control ALP also in these invertebrates.

The high degree of conservation of the MiTF/TFE transcription factors across evolution should be exploited for the research of modulators of these proteins. The possibility of being inspired not only of many classical model organisms but also of several non-canonical animal models may be very relevant to implement the in vivo characterization of these factors and represent very good tools to analyze in detail different aspects of the MiTF/TFE transcription factors that are still unclear.

## Conclusions and Future Directions

Autophagy dysfunction has been described in different neurodegenerative disorders. All the members of the MiTF/TFE family have been shown to participate in the regulation of autophagy, and in other processes that are relevant for brain physiology. However, many aspects related to the basic biology of these transcription factors remain unknown. For instance, MiTF/TFE transcription factors can form both homodimers and heterodimers with any other family member, but little is known about the functional difference between homodimers and heterodimers. Furthermore, with the exception of TFE3, all family members have alternative transcripts which display different tissue distribution patterns, and it remains to be determined the functional importance of these transcripts and whether this may result in cell-type–specific regulatory networks. As MiTF/TFE transcription factors are conserved across species, comparing MiTF/TFE protein function and regulation in different and appropriate animal models may provide a better understanding of their physiological function in the CNS. Moreover, the use of different model organisms may provide a valuable tool for understanding the roles of these transcription factors common to all forms of eukaryotic life and how their impairment may be implicated in neurodegeneration. Thus, it appears fundamental to decipher the factors that are responsible for MiTF/TFE transcription factor regulation and their interplay. This would allow modulating autophagy and other relevant pathways for brain cells via MiTF/TFE family members in a tissue/cell-specific manner thus avoiding negative side effects.

## Data Availability

Not applicable.

## References

[1] Perera RM, Di Malta C, Ballabio A (2019). MiT/TFE family of transcription factors, lysosomes, and cancer. Annu Rev Cancer Biol.

[2] Medina DL, Di PS, Peluso I (2016). Lysosomal calcium signaling regulates autophagy via calcineurin and TFEB. Nat Cell Biol.

[3] Palmisano NJ, Meléndez A, Kelly AL (2015). A protein conjugation system essential for autophagy. Nature.

[4] Hemesath TJ, Steingrímsson E, McGill G (1994). Microphthalmia, a critical factor in melanocyte development, defines a discrete transcription factor family. Genes Dev.

[5] Sato S, Roberts K, Gambino G (1997). CBP/p300 as a co-factor for the microphthalmia transcription factor. Oncogene.

[6] Atacho DAM, Reynisson H, Petursdottir AT, et al (2020) Mitf links neuronal activity and long-term homeostatic intrinsic plasticity. Eneuro 7:ENEURO.0412–19.2020. 10.1523/ENEURO.0412-19.2020 PMC7174873 32193365

[7] Park K, Lim H, Kim J (2022). Lysosomal Ca2+-mediated TFEB activation modulates mitophagy and functional adaptation of pancreatic β-cells to metabolic stress. Nat Commun.

[8] Yu S, Wang Z, Ding L, Yang L (2020). The regulation of TFEB in lipid homeostasis of non-alcoholic fatty liver disease: molecular mechanism and promising therapeutic targets. Life Sci.

[9] Li M, Wang Z, Wang P (2021). TFEB: a emerging regulator in lipid homeostasis for atherosclerosis. Front Physiol.

[10] Kim HJ, Joe Y, Rah SY, et al (2018) Carbon monoxide-induced TFEB nuclear translocation enhances mitophagy/mitochondrial biogenesis in hepatocytes and ameliorates inflammatory liver injury. Cell Death Dis 9:. 10.1038/s41419-018-1112-x PMC6193007 30333475

[11] Parzych KR, Klionsky DJ (2013). An overview of autophagy: morphology, mechanism, and regulation. Antioxid Redox Signal.

[12] Yang J, Chai X, Zhao X-X, Li X (2017). Comparative genomics revealed the origin and evolution of autophagy pathway. J Syst Evol.

[13] Hughes T, Rusten TE (2007). Origin and evolution of self-consumption : autophagy. Adv Exp Med Biol.

[14] Mindell JA (2012). Lysosomal acidification mechanisms. Annu Rev Physiol.

[15] Loos B, Du Toit A, Hofmeyr JHS (2014). Defining and measuring autophagosome flux - concept and reality. Autophagy.

[16] Martini-Stoica H, Xu Y, Ballabio A, Zheng H (2017). The autophagy–lysosomal pathway in neurodegeneration: a TFEB perspective. Trends Neurosci.

[17] Fujikake N, Shin M, Shimizu S (2018). Association between autophagy and neurodegenerative diseases. Front Neurosci.

[18] Medina DL, Fraldi A, Bouche V (2011). Transcriptional activation of lysosomal exocytosis promotes cellular clearance. Dev Cell.

[19] Polito VA, Li H, Martini-Stoica H (2014). Selective clearance of aberrant tau proteins and rescue of neurotoxicity by transcription factor EB. EMBO Mol Med.

[20] Soldati C, Lopez-Fabuel I, Wanderlingh LG (2021). Repurposing of tamoxifen ameliorates CLN3 and CLN7 disease phenotype. EMBO Mol Med.

[21] Lapierre LR, De Magalhaes Filho CD, McQuary PR (2013). The TFEB orthologue HLH-30 regulates autophagy and modulates longevity in Caenorhabditis elegans. Nat Commun.

[22] Bouché V, Espinosa AP, Leone L (2016). Drosophila Mitf regulates the V-ATPase and the lysosomal-autophagic pathway. Autophagy.

[23] Cunningham KM, Maulding K, Ruan K (2020). Tfeb/mitf links impaired nuclear import to autophagolysosomal dysfunction in c9-als. Elife.

[24] Arotcarena ML, Bourdenx M, Dutheil N (2019). Transcription factor EB overexpression prevents neurodegeneration in experimental synucleinopathies. JCI Insight.

[25] Steingrímsson E, Tessarollo L, Reid SW (1998). The bHLH-Zip transcription factor Tfeb is essential for placental vascularization. Development.

[26] Steingrímsson E, Copeland NG, Jenkins NA (2004). Melanocytes and the microphthalmia transcription factor network. Annu Rev Genet.

[27] Zhao GQ, Zhao Q, Zhou X (1993). TFEC, a basic helix-loop-helix protein, forms heterodimers with TFE3 and inhibits TFE3-dependent transcription activation. Mol Cell Biol.

[28] Sardiello M, Palmieri M, di Ronza A (2009). A gene network regulating lysosomal biogenesis and function. Science.

[29] Palmieri M, Impey S, Kang H (2011). Characterization of the CLEAR network reveals an integrated control of cellular clearance pathways. Hum Mol Genet.

[30] Aksan I, Goding CR (1998). Targeting the microphthalmia basic helix-loop-helix–leucine zipper transcription factor to a subset of E-box elements in vitro and in vivo. Mol Cell Biol.

[31] Pogenberg V, Ögmundsdóttir MH, Bergsteinsdóttir K (2012). Restricted leucine zipper dimerization and specificity of DNA recognition of the melanocyte master regulator MITF. Genes Dev.

[32] Steingrímsson E, Tessarollo L, Pathak B (2002). Mitf and Tfe3, two members of the Mitf-Tfe family of bHLH-Zip transcription factors, have important but functionally redundant roles in osteoclast development. Proc Natl Acad Sci U S A.

[33] Byun S, Seok S, Kim YC (2020). Fasting-induced FGF21 signaling activates hepatic autophagy and lipid degradation via JMJD3 histone demethylase. Nat Commun.

[34] Seok S, Fu T, Choi SE (2014). Transcriptional regulation of autophagy by an FXR-CREB axis. Nature.

[35] Song W, Zhang CL, Gou L (2019). Endothelial TFEB (transcription factor EB) restrains IKK (IκB kinase)-p65 pathway to attenuate vascular inflammation in diabetic db/db mice. Arterioscler Thromb Vasc Biol.

[36] Settembre C, Ballabio A (2011). TFEB regulates autophagy: an integrated coordination of cellular degradation and recycling processes. Autophagy.

[37] Kuiper RP, Schepens M, Thijssen J (2004). Regulation of the MiTF/TFE bHLH-LZ transcription factors through restricted spatial expression and alternative splicing of functional domains. Nucleic Acids Res.

[38] La Spina M, Contreras PS, Rissone A (2021). MiT/TFE family of transcription factors: an evolutionary perspective. Front Cell Dev Biol.

[39] Settembre C, Polito VA, Garcia M (2013). TFEB links autophagy to lysosomal biogenesis carmine. Science.

[40] Napolitano G, Ballabio A (2016). TFEB at a glance. J Cell Sci.

[41] Zoncu R, Bar-Peled L, Efeyan A (2011). mTORC1 senses lysosomal amino acids through an inside-out mechanism that requires the Vacuolar H+-ATPase. Science.

[42] Sancak Y, Bar-Peled L, Zoncu R (2010). Ragulator-rag complex targets mTORC1 to the lysosomal surface and is necessary for its activation by amino acids. Cell.

[43] Rabanal-Ruiz Y, Korolchuk VI (2018). mTORC1 and nutrient homeostasis: the central role of the lysosome. Int J Mol Sci.

[44] Inoki K, Ouyang H, Zhu T (2006). TSC2 integrates Wnt and energy signals via a coordinated phosphorylation by AMPK and GSK3 to regulate cell growth. Cell.

[45] Paquette M, El-Houjeiri L, Zirden LC (2021). AMPK-dependent phosphorylation is required for transcriptional activation of TFEB and TFE3. Autophagy.

[46] Benito-Cuesta I, Ordóñez-Gutiérrez L, Wandosell F (2021). AMPK activation does not enhance autophagy in neurons in contrast to MTORC1 inhibition: different impact on β-amyloid clearance. Autophagy.

[47] Li C, Wang X, Li X (2019). Proteasome inhibition activates autophagy-lysosome pathway associated with TFEB dephosphorylation and nuclear translocation. Front Cell Dev Biol.

[48] Sha Y, Rao L, Settembre C (2017). STUB 1 regulates TFEB -induced autophagy–lysosome pathway. EMBO J.

[49] Rao L, Sha Y, Eissa NT (2017). The E3 ubiquitin ligase STUB1 regulates autophagy and mitochondrial biogenesis by modulating TFEB activity. Mol Cell Oncol.

[50] Amae S, Fuse N, Yasumoto KI (1998). Identification of a novel isoform of microphthalmia-associated transcription factor that is enriched in retinal pigment epithelium. Biochem Biophys Res Commun.

[51] Udono T, Yasumoto KI, Takeda K (2000). Structural organization of the human microphthalmia-associated transcription factor gene containing four alternative promoters. Biochim Biophys Acta - Gene Struct Expr.

[52] Fuse N, Yasumoto K, Takeda K (1999). Molecular cloning of cDNA encoding a novel microphthalmia-associated transcription factor isoform with a distinct amino-terminus. J Biochem.

[53] Takeda K, Yasumoto KI, Kawaguchi N (2002). Mitf-D, a newly identified isoform, expressed in the retinal pigment epithelium and monocyte-lineage cells affected by Mitf mutations. Biochim Biophys Acta - Gene Struct Expr.

[54] Oboki K, Morii E, Kataoka TR (2002). Isoforms of mi transcription factor preferentially expressed in cultured mast cells of mice. Biochem Biophys Res Commun.

[55] Aberdam D, Galliano M, Vaillyl J (1994). Herlitz's junctional epidermolysis bullosa is linked to mutations in the gene (LAMC2) for the γ2 subunit of nicein/kalinin (LAMININ–5). Nat Genet.

[56] Hershey CL, Fisher DE (2005). Genomic analysis of the microphthalmia locus and identification of the MITF-J/Mitf-J isoform. Gene.

[57] Takemoto CM, Yoon YJ, Fisher DE (2002). The identification and functional characterization of a novel mast cell isoform of the microphthalmia-associated transcription factor. J Biol Chem.

[58] Hodgkinson CA, Moore KJ, Nakayama A (1993). Mutations at the mouse microphthalmia locus are associated with defects in a gene encoding a novel basic-helix-loop-helix-zipper protein. Cell.

[59] Martina JA, Diab HI, Lishu L (2014). The nutrient-responsive transcription factor TFE3 promotes autophagy, lysosomal biogenesis, and clearance of cellular debris. Sci Signal.

[60] Ohba K, Takeda K, Yamamoto H, Shibahara S (2015). Microphthalmia-associated transcription factor is expressed in projection neurons of the mouse olfactory bulb. Genes Cells.

[61] Lee YC, Durr A, Majczenko K (2012). Mutations in KCND3 cause spinocerebellar ataxia type 22. Ann Neurol.

[62] Duarri A, Jezierska J, Fokkens M (2012). Mutations in potassium channel KCND3 cause spinocerebellar ataxia type 19. Ann Neurol.

[63] Tachibana M (2000). MITF: a stream flowing for pigment cells. Pigment Cell Res.

[64] Yonashiro R, Sugiura A, Miyachi M (2009). Mutant SOD1 and attenuates mutant SOD1-induced reactive oxygen species generation. Mol Biol Cell.

[65] Maruotti J, Thein T, Zack DJ, Esumi N (2012). MITF-M, a “melanocyte-specific” isoform, is expressed in the adult retinal pigment epithelium. Pigment Cell Melanoma Res.

[66] Haq R, Fisher DE (2011). Biology and clinical relevance of the micropthalmia family of transcription factors in human cancer. J Clin Oncol.

[67] Haq R, Shoag J, Andreu-Perez P (2013). Oncogenic BRAF regulates oxidative metabolism via PGC1α and MITF. Cancer Cell.

[68] Goding CR, Arnheiter H (2019). MITF - the first 25 years. Genes Dev.

[69] Lamprecht R (2021). Actin cytoskeleton role in the maintenance of neuronal morphology and long-term memory. Cells.

[70] Civiero L, Greggio E (2018). PAKs in the brain: function and dysfunction. Biochim Biophys Acta - Mol Basis Dis.

[71] Möller K, Sigurbjornsdottir S, Arnthorsson AO (2019). MITF has a central role in regulating starvation-induced autophagy in melanoma. Sci Rep.

[72] Pang X, Zheng X, Kong X (2019). A homozygous MITF mutation leads to familial Waardenburg syndrome type 4. Am J Med Genet Part A.

[73] Smith SD, Kelley PM, Kenyon JB, Hoover D (2000). Tietz syndrome (hypopigmentation/deafness) caused by mutation of MITF. J Med Genet.

[74] Sun J, Hao Z, Luo H (2017). Functional analysis of a nonstop mutation in MITF gene identified in a patient with Waardenburg syndrome type 2. J Hum Genet.

[75] George A, Zand DJ, Hufnagel RB (2016). Biallelic mutations in MITF cause coloboma, osteopetrosis, microphthalmia, macrocephaly, albinism, and deafness. Am J Hum Genet.

[76] Ferron M, Shimazu J, Karsenty G (2013). A RANKL-PKCβ-TFEB signaling cascade is necessary for lysosomal biogenesis in osteoclasts. Genes Dev.

[77] Settembre C, De Cegli R, Mansueto G (2013). TFEB controls cellular lipid metabolism through a starvation-induced autoregulatory loop. Nat Cell Biol.

[78] Erlich AT, Brownlee DM, Beyfuss K, Hood DA (2018). Exercise induces TFEB expression and activity in skeletal muscle in a pgc-1α-dependent manner. Am J Physiol - Cell Physiol.

[79] Mansueto G, Armani A, Viscomi C (2017). Transcription factor EB controls metabolic flexibility during exercise. Cell Metab.

[80] Pastore N, Brady OA, Diab HI (2016). TFEB and TFE3 cooperate in the regulation of the innate immune response in activated macrophages. Autophagy.

[81] Raben N, Puertollano R (2016). TFEB and TFE3, linking lysosomes to cellular adaptation to stress. Annu Rev Cell Dev Biol.

[82] Martina JA, Diab HI, Brady OA, Puertollano R (2016). TFEB and TFE 3 are novel components of the integrated stress response. EMBO J.

[83] Taniguchi M, Nadanaka S, Tanakura S (2015). TFE3 is a BHLH-ZIP-type transcription factor that regulates the mammalian Golgi stress response. Cell Struct Funct.

[84] Walker M, Kublin JG, Zunt JR (2013). Neuronal ER stress in axon injury and neurodegeneration. Ann Neurol.

[85] Alvarez-Miranda EA, Sinnl M, Farhan H (2015). Alteration of Golgi structure by stress: a link to neurodegeneration?. Front Neurosci.

[86] Smith M, Wilkinson S (2017). ER homeostasis and autophagy. Essays Biochem.

[87] Deng S, Liu J, Wu X, Lu W (2020). Golgi apparatus: a potential therapeutic target for autophagy-associated neurological diseases. Front Cell Dev Biol.

[88] Rehli M, Lichanska A, Cassady AI (1999). TFEC is a macrophage-restricted member of the microphthalmia-TFE subfamily of basic helix-loop-helix leucine zipper transcription factors. J Immunol.

[89] Rehli M, Den Elzen N, Cassady AI (1999). Cloning and characterization of the murine genes for bHlH-ZIP transcription factors TFEC and TFEB reveal a common gene organization for all MiT subfamily members. Genomics.

[90] Rehli M, Sulzbacher S, Pape S (2005). Transcription factor Tfec contributes to the IL-4-inducible expression of a small group of genes in mouse macrophages including the granulocyte colony-stimulating factor receptor. J Immunol.

[91] Cai Q, Ganesan D (2022). Regulation of neuronal autophagy and the implications in neurodegenerative diseases. Neurobiol Dis.

[92] Mizushima N, Levine B, Cuervo AM, Klionsky DJ (2008). Autophagy fights disease through cellular self-digestion. Nature.

[93] Streubel-Gallasch L, Giusti V, Sandre M (2021). Parkinson’s disease–associated LRRK2 interferes with astrocyte-mediated alpha-synuclein clearance. Mol Neurobiol.

[94] Zhu Z, Yang C, Iyaswamy A (2019). Balancing mTOR signaling and autophagy in the treatment of Parkinson’s disease. Int J Mol Sci.

[95] Cortes CJ, La Spada AR (2014). The many faces of autophagy dysfunction in Huntington’s disease: from mechanism to therapy. Drug Discov Today.

[96] Zhang Z, Yang X, Song YQ, Tu J (2021). Autophagy in Alzheimer’s disease pathogenesis: therapeutic potential and future perspectives. Ageing Res Rev.

[97] Pircs K, Drouin-Ouellet J, Horváth V, et al (2021) Distinct subcellular autophagy impairments in induced neurons from Huntington’s disease patients. Brain awab473. 10.1093/brain/awab473 PMC9473361 34936701

[98] Choi I, Zhang Y, Seegobin SP (2020). Microglia clear neuron-released α-synuclein via selective autophagy and prevent neurodegeneration. Nat Commun.

[99] Binder JL, Chander P, Deretic V (2020). Optical induction of autophagy via transcription factor EB (TFEB) reduces pathological tau in neurons. PLoS ONE.

[100] Nascimento AC, Erustes AG, Reckziegel P (2020). α-Synuclein overexpression induces lysosomal dysfunction and autophagy impairment in human neuroblastoma SH-SY5Y. Neurochem Res.

[101] Feng Q, Luo Y, Zhang XN (2020). MAPT/Tau accumulation represses autophagy flux by disrupting IST1-regulated ESCRT-III complex formation: a vicious cycle in Alzheimer neurodegeneration. Autophagy.

[102] Manczak M, Kandimalla R, Yin X, Reddy PH (2018). Hippocampal mutant APP and amyloid beta-induced cognitive decline, dendritic spine loss, defective autophagy, mitophagy and mitochondrial abnormalities in a mouse model of Alzheimer’s disease. Hum Mol Genet.

[103] Pircs K, Petri R, Madsen S (2018). Huntingtin aggregation impairs autophagy, leading to argonaute-2 accumulation and global microRNA dysregulation. Cell Rep.

[104] Komatsu M, Waguri S, Chiba T (2006). Loss of autophagy in the central nervous system causes neurodegeneration in mice. Nature.

[105] Hara T, Nakamura K, Matsui M (2006). Suppression of basal autophagy in neural cells causes neurodegenerative disease in mice. Nature.

[106] Wang H, Wang R, Xu S, Lakshmana MK (2016). Transcription factor EB Is selectively reduced in the nuclear fractions of Alzheimer’s and amyotrophic lateral sclerosis brains. Neurosci J.

[107] Decressac M, Mattsson B, Weikop P (2013). TFEB-mediated autophagy rescues midbrain dopamine neurons from α-synuclein toxicity. Proc Natl Acad Sci U S A.

[108] Plotegher N, Kumar D, Tessari I (2014). The chaperone-like protein 14-3-3h interacts with human a-synuclein aggregation intermediates rerouting the amyloidogenic pathway and reducing a-synuclein cellular toxicity. Hum Mol Genet.

[109] Tiribuzi R, Crispoltoni L, Porcellati S (2014). MiR128 up-regulation correlates with impaired amyloid β(1–42) degradation in monocytes from patients with sporadic Alzheimer’s disease. Neurobiol Aging.

[110] Reddy K, Cusack CL, Nnah IC (2016). Dysregulation of nutrient sensing and CLEARance in presenilin deficiency. Cell Rep.

[111] Guo X, Tang P, Chen L (2017). Amyloid β-induced redistribution of transcriptional factor EB and lysosomal dysfunction in primary microglial cells. Front Aging Neurosci.

[112] Coffey EE, Beckel JM, Laties AM, Mitchell CH (2014). Lysosomal alkalization and dysfunction in human fibroblasts with the Alzheimer’s disease-linked presenilin 1 A246E mutation can be reversed with cAMP. Neuroscience.

[113] Zhang X, Garbett K, Veeraraghavalu K (2012). A role for presenilins in autophagy revisited: normal acidification of lysosomes in cells lacking PSEN1 and PSEN2. J Neurosci.

[114] Bordi M, Berg MJ, Mohan PS (2016). Autophagy flux in CA1 neurons of Alzheimer hippocampus: increased induction overburdens failing lysosomes to propel neuritic dystrophy. Autophagy.

[115] Zhang H, Alsaleh G, Feltham J (2019). Polyamines control eIF5A hypusination, TFEB translation, and autophagy to reverse B cell senescence. Mol Cell.

[116] Kielian T (2019). Lysosomal storage disorders: pathology within the lysosome and beyond. J Neurochem.

[117] Seranova E, Connolly KJ, Zatyka M (2017). Dysregulation of autophagy as a common mechanism in lysosomal storage diseases. Essays Biochem.

[118] Awad O, Sarkar C, Panicker LM (2015). Altered TFEB-mediated lysosomal biogenesis in Gaucher disease iPSC-derived neuronal cells. Hum Mol Genet.

[119] Brown RA, Voit A, Srikanth MP (2019). mTOR hyperactivity mediates lysosomal dysfunction in Gaucher’s disease iPSC-neuronal cells. DMM Dis Model Mech.

[120] Khan SA, Peracha H, Ballhausen D (2018). Molecular genetics and metabolism. Mol Genet Metab.

[121] Spampanato C, Feeney E, Li L (2013). Transcription factor EB (TFEB) is a new therapeutic target for Pompe disease. EMBO Mol Med.

[122] van Kooten HA, Roelen CHA, Brusse E (2021). Cardiovascular disease in non-classic Pompe disease: a systematic review. Neuromuscul Disord.

[123] Martina JA, Diab HI, Lishu L (2004). The nutrient-responsive transcription factor TFE3, promotes autophagy, lysosomal biogenesis, and clearance of cellular debris. Sci Signal.

[124] Wang H, Wang R, Carrera I (2016). TFEB overexpression in the P301S model of tauopathy mitigates increased PHF1 levels and lipofuscin puncta and rescues memory deficits. eNeuro.

[125] Xu Y, Du S, Marsh JA (2020). TFEB regulates lysosomal exocytosis of tau and its loss of function exacerbates tau pathology and spreading. Mol Psychiatry.

[126] Bécot A, Pardossi-Piquard R, Bourgeois A (2020). The transcription factor EB reduces the intraneuronal accumulation of the beta-secretase-derived APP fragment C99 in cellular and mouse Alzheimer’s disease models. Cells.

[127] Xiao Q, Yan P, Ma X (2014). Enhancing astrocytic lysosome biogenesis facilitates Aβ clearance and attenuates amyloid plaque pathogenesis. J Neurosci.

[128] Martini-Stoica H, Cole AL, Swartzlander DB (2018). TFEB enhances astroglial uptake of extracellular tau species and reduces tau spreading. J Exp Med.

[129] Vodicka P, Chase K, Iuliano M (2016). Autophagy activation by transcription factor EB (TFEB) in striatum of HD Q175 / Q7 mice. J Hungtintons Dis.

[130] Brattås PL, Hersbach BA, Madsen S (2020). Impact of differential and time-dependent autophagy activation on therapeutic efficacy in a model of Huntington disease. Autophagy.

[131] Napolitano G, Di Malta C, Esposito A (2020). A substrate-specific mTORC1 pathway underlies Birt–Hogg–Dubé syndrome. Nature.

[132] Song W, Wang F, Lotfi P (2014). 2-Hydroxypropyl-β-cyclodextrin promotes transcription factor EB-mediated activation of autophagy: Implications for therapy. J Biol Chem.

[133] Kilpatrick K, Zeng Y, Hancock T, Segatori L (2015). Genetic and chemical activation of TFEB mediates clearance of aggregated α-synuclein. PLoS ONE.

[134] Chandra S, Jana M, Pahan K (2018). Aspirin induces lysosomal biogenesis and attenuates amyloid plaque pathology in a mouse model of Alzheimer’s disease via PPARα. J Neurosci.

[135] Song W, Lee SS, Savini M (2014). Ceria nanoparticles stabilized by organic surface coatings activate the lysosome-autophagy system and enhance autophagic clearance. ACS Nano.

[136] Design D, Gao L, Li X (2020). Chlorogenic acid alleviates a β 25–35 -induced autophagy and cognitive impairment via the mTOR/TFEB signaling pathway. Drug Des Devel Ther.

[137] Chandra S, Roy A, Jana M, Pahan K (2019). Cinnamic acid activates PPARα to stimulate lysosomal biogenesis and lower amyloid plaque pathology in an Alzheimer’s disease mouse model. Neurobiol Dis.

[138] Song JX, Malampati S, Zeng Y (2020). A small molecule transcription factor EB activator ameliorates beta-amyloid precursor protein and Tau pathology in Alzheimer’s disease models. Aging Cell.

[139] Zhuang XX, Wang SF, Tan Y (2020). Pharmacological enhancement of TFEB-mediated autophagy alleviated neuronal death in oxidative stress-induced Parkinson’s disease models. Cell Death Dis.

[140] Wang Z, Yang C, Liu J (2020). A curcumin derivative activates TFEB and protects against parkinsonian neurotoxicity in vitro. Int J Mol Sci.

[141] Chen Y, Xu S, Wang N (2019). Dynasore suppresses mTORC1 activity and induces autophagy to regulate the clearance of protein aggregates in neurodegenerative diseases. Neurotox Res.

[142] Kim S, Choi KJ, Cho S (2016). Fisetin stimulates autophagic degradation of phosphorylated tau via the activation of TFEB and Nrf2 transcription factors. Sci Rep.

[143] Chauhan S, Ahmed Z, Bradfute SB (2015). Processes for activation of autophagy with. Nat Commun.

[144] Moskot M, Montefusco S, Jako J (2014). The phytoestrogen genistein modulates lysosomal metabolism and transcription factor EB (TFEB) Activation. J Biol Chem.

[145] Parr C, Carzaniga R, Gentleman SM (2012). Glycogen synthase kinase 3 inhibition promotes lysosomal biogenesis and autophagic degradation of the amyloid- precursor protein. Mol Cell Biol.

[146] Meng X, Luo Y, Liang T (2016). Gypenoside XVII enhances lysosome biogenesis and autophagy flux and accelerates autophagic clearance of amyloid-β through TFEB activation.

[147] Chen Y, Wang H, Ying Z, Gao Q (2020). Ibudilast enhances the clearance of SOD1 and TDP-43 aggregates through TFEB-mediated autophagy and lysosomal biogenesis: the new molecular mechanism of ibudilast and its implication for neuroprotective therapy. Biochem Biophys Res Commun.

[148] Song H, Vladimirov A, Kim N, Kim D (2019). Molecular and cellular neuroscience ouabain activates transcription factor EB and exerts neuroprotection in models of Alzheimer ’ s disease. Mol Cell Neurosci.

[149] Yao XC, Xue X, Zhang HT (2019). Pseudoginsenoside-F11 alleviates oligomeric β amyloid-induced endosome-lysosome defects in microglia. Traffic.

[150] Palmieri M, Pal R, Nelvagal HR (2017). mTORC1-independent TFEB activation via Akt inhibition promotes cellular clearance in neurodegenerative storage diseases. Nat Commun.

[151] Rusmini P, Cortese K, Crippa V (2019). Trehalose induces autophagy via lysosomal-mediated TFEB activation in models of motoneuron degeneration. Autophagy.

[152] Martins WK, do Nascimento da Silva M, Pandey K (2021). Autophagy-targeted therapy to modulate age-related diseases: success, pitfalls, and new directions. Curr Res Pharmacol Drug Discov.

[153] Capuozzo A, Montefusco S, Cacace V (2022). Fluoxetine ameliorates mucopolysaccharidosis type IIIA. Mol Ther.

[154] Kauffman EC, Ricketts CJ, Rais-Bahrami S (2014). Molecular genetics and cellular features of TFE3 and TFEB fusion kidney cancers. Nat Rev Urol.

[155] Liang J, Jia X, Wang K, Zhao N (2018). High expression of TFEB is associated with aggressive clinical features in colorectal cancer. Onco Targets Ther.

[156] Slade L, Pulinilkunnil T (2017). The MiTF/TFE family of transcription factors: master regulators of organelle signaling, metabolism, and stress adaptation. Mol Cancer Res.

[157] Alesi N, Akl EW, Khabibullin D (2021). TSC2 regulates lysosome biogenesis via a non-canonical RAGC and TFEB-dependent mechanism. Nat Commun.

[158] Frézal L, Félix MA (2015). C. elegans outside the Petri dish. Elife.

[159] Franco-Juárez B, Mejía-Martínez F, Moreno-Arriola E (2018). A high glucose diet induces autophagy in a HLH-30/TFEB-dependent manner and impairs the normal lifespan of C. elegans. Aging (Albany NY).

[160] Wani KA, Goswamy D, Taubert S (2021). Nhr-49/ppar-a and hlh-30/tfeb cooperate for C. elegans host defense via a flavin-containing monooxygenase. Elife.

[161] Dall KB, Havelund JF, Harvald EB (2021). HLH-30-dependent rewiring of metabolism during starvation in C. elegans. Aging Cell.

[162] Chang JT, Kumsta C, Hellman AB (2017). Spatiotemporal regulation of autophagy during Caenorhabditis elegans aging. Elife.

[163] Cooper JF, Van Raamsdonk JM (2018). Modeling Parkinson’s disease in C. elegans. J Parkinsons Dis.

[164] Lapierre LR, De Magalhases Filho CD, Mcquary PR (2013). The TFEB orthologue HLH-30 regulates autophagy and modulates longevity in Caenorhabditis elegans. Nat Commun.

[165] Tolwinski NS (2017). Introduction: Drosophila-a model system for developmental biology. J Dev Biol.

[166] De Lazzari F, Bisaglia M, Zordan MA, Sandrelli F (2018). Circadian rhythm abnormalities in Parkinson’s disease from humans to flies and back. Int J Mol Sci.

[167] Hirth F (2012). Drosophila melanogaster in the study of human neurodegeneration. CNS Neurol Disord - Drug Targets.

[168] Hallsson JH, Haflidadóttir BS, Stivers C (2004). The basic helix-loop-helix leucine zipper transcription factor Mitf is conserved in Drosophila and functions in eye development. Genetics.

[169] Xi Y, Noble S, Ekker M (2011). Modeling neurodegeneration in zebrafish. Curr Neurol Neurosci Rep.

[170] Lister JA, Close J, Raible DW (2001). Duplicate mitf genes in zebrafish: complementary expression and conservation of melanogenic potential. Dev Biol.

[171] Meireles AM, Shen K, Zoupi L (2019). The lysosomal transcription factor TFEB represses myelination downstream of the Rag-Ragulator complex. Dev Cell.

[172] Mahony CB, Fish RJ, Pasche C, Bertrand JY (2016). Tfec controls the hematopoietic stem cell vascular niche during zebrafish embryogenesis. Blood.

[173] Russo R, Chiaramonte M, Lampiasi N, Zito F (2019). MITF: an evolutionarily conserved transcription factor in the sea urchin Paracentrotus lividus. Genetica.

[174] Mao J, Zhang X, Zhang W (2019). Genome-wide identification, characterization and expression analysis of the MITF gene in Yesso scallops (Patinopecten yessoensis) with different shell colors. Gene.

